# *Salvia miltiorrhiza* Bunge root in the treatment of myocardial fibrosis: research progress and challenges

**DOI:** 10.3389/fphar.2025.1554696

**Published:** 2025-03-31

**Authors:** Qianrong Li, Chunzhen Ren, Bing Jiang, Xuehan Wang, Chunling Wang, Xiaodong Zhi, Linchan Li, Xiaoying Guo, Xinke Zhao, Yingdong Li

**Affiliations:** ^1^ School of Traditional Chinese and Western Medicine, Gansu University of Chinese Medicine, Lanzhou, China; ^2^ College of Integrated Traditional Chinese and Western Medicine, Gansu Province Key Laboratory of Chinese Medicine for the Prevention and Treatment of Chronic Diseases, Gansu University of Chinese Medicine, Lanzhou, China; ^3^ Key Clinical Specialty of the National Health Commission of the People’s Republic of China, Key Specialized Cardiovascular Laboratory National Administration of Traditional Chinese Medicine, Lanzhou, China; ^4^ The First Clinical Medical College, Lanzhou University, Lanzhou, China; ^5^ Affiliated Hospital of Gansu University of Chinese Medicine, Lanzhou, China; ^6^ Oncology Department, Shaanxi Provincial Hospital of Chinese Medicine, Xi’an, China

**Keywords:** natural medicine *Salvia miltiorrhiza* Bunge, MF, advancements in research, metabolites, pharmacological mechanism

## Abstract

Myocardial fibrosis (MF) involves the activation and excessive proliferation of cardiac fibroblasts (CFs) in the extracellular matrix, leading to increased collagen expression that impairs cardiac function. Currently, there are no effective pharmacological treatments for MF. Traditional Chinese Medicine (TCM), particularly *Salvia miltiorrhiza* Bunge [Lamiaceae; *Salviae miltiorrhizae* radix et rhizoma], has gained attention for its potential in treating MF. Recent studies indicate significant therapeutic effects of its active metabolites, supporting its use in MF treatment and positioning it as a promising candidate for drug development. Aim of the review: This article reviews the research and mechanisms of *S. miltiorrhiza*’s effective metabolites and preparations in treating MF, providing a reference for future clinical treatments. A systematic literature search was conducted in PubMed, Web of Science, CNKI, and Google Scholar (January 2000–October 2024) using keywords: “myocardial fibrosis,” “cardiac fibrosis,” “*Salvia miltiorrhiza* Bunge,” “extract,” and “botanical drug.” Results: The active metabolites of *S. miltiorrhiza* and its metabolite preparations exert anti-fibrotic effects through pleiotropic mechanisms, including suppression of ventricular remodeling, modulation of autophagy, inhibition of oxidative stress and cardiomyocyte apoptosis, and regulation of extracellular matrix homeostasis and immune-inflammatory responses. Conclusion: Research indicates that *S. miltiorrhiza* is beneficial for managing MF, but further studies are needed to identify its chemical metabolites and regulatory mechanisms. Large-scale, multi-center clinical trials are also necessary to assess treatment safety. This review offers insights for developing new anti-MF pharmacotherapies.

## Graphical Abstract

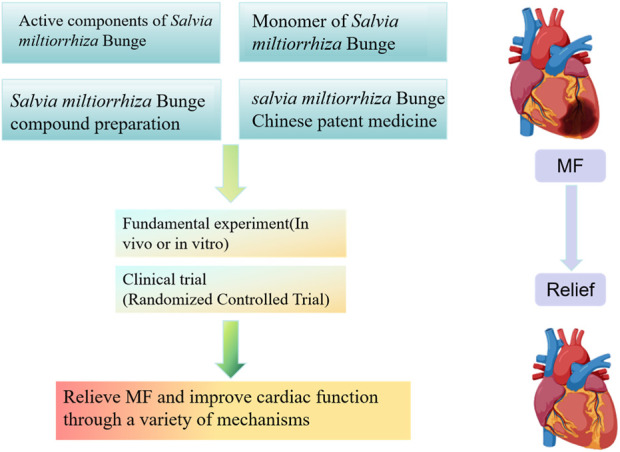

## 1 Introduction

Myocardial fibrosis (MF) is a common pathological mechanism linked to several cardiovascular disorders, including myocardial infarction, hypertensive heart disease, and dilated cardiomyopathy. This condition is characterized by an abnormal accumulation of extracellular matrix metabolites, along with the excessive proliferation and activation of cardiac fibroblasts (CFs). Thus, there is a significant increase in collagen fiber deposition, collagen content, and overall volume of collagen. These pathological changes contribute to a decrease in myocardial compliance and cardiac function, which may lead to arrhythmias and sudden cardiac death (Maruyama and Imanaka-Yoshida, 2022). Globally, myocardial fibrosis is prevalent in 30%–50% of heart failure patients and is associated with a 2.3-fold increase in all-cause mortality due to its adverse prognostic implications (Behnoush et al., 2023). MF is characterized by rapid progression, high mortality, and multifactorial pathogenesis involving inflammatory, oxidative, and apoptotic pathways. Contemporary medical understanding indicates that MF arises from a single factor and the interplay of elements such as inflammatory responses, oxidative stress, the renin-angiotensin-aldosterone system (RAAS), matrix metalloproteinases (MMPs), growth factors, noncoding RNAs, endothelial dysfunction, and cardiomyocyte apoptosis and necrosis. The clinical diagnosis of myocardial fibrosis faces significant challenges. Endomyocardial biopsy, due to its invasiveness, is difficult to widely implement. Meanwhile, emerging non-invasive imaging techniques such as cardiac magnetic resonance (CMR) T1 mapping and extracellular volume (ECV) quantification have significantly improved the detection rate (with a sensitivity of up to 89%). However, their standardized application is still limited by the availability of equipment and cost (Bengel et al., 2023). The pharmacological agents used in clinical treatment include statins, angiotensin-converting enzyme inhibitors (ACEI), angiotensin II receptor antagonists (ARBs), angiotensin receptor-neprilysin inhibitors (ARNIs), sodium-glucose cotransporter 2 inhibitors (SGLT2i), and aldosterone receptor antagonists. Although these medications can alleviate symptoms, their efficacy in preventing or reversing MF is limited (Liu et al., 2023). Discontinuing medication or developing tolerance can worsen a patient’s condition, highlighting the urgent need for effective and safe pharmacological interventions for MF.

*Salvia miltiorrhiza* Bunge [Lamiaceae; *Salviae miltiorrhizae* radix et rhizoma], a perennial botanical drug deeply rooted in traditional Chinese medicine (TCM), has been revered for centuries as a cornerstone therapy for cardiovascular ailments (Wu et al., 2022). Its historical applications, documented in classical texts such as Shennong Bencao Jing, emphasize its efficacy in “promoting blood circulation, resolving blood stasis, and relieving pain”—principles that align with modern understandings of pathologies marked by microcirculatory dysfunction and fibrotic remodeling, such as MF (Li Q. et al., 2022; Qi et al., 2024; Wang et al., 2021). The term MF does not appear in TCM. However, it can be classified under the category of “chest pain and heartache” within this medical framework. Contemporary studies link its pathogenesis to a deficiency of the fundamental essence and an excess of superficial conditions, including blood stasis, phlegm turbidity, and heat toxins (Ren et al., 2022). Recent studies have focused on the use of *S. miltiorrhiza* for the prevention and treatment of MF, demonstrating its protective effects on cardiac health. *S. miltiorrhiza* has emerged as a promising candidate, offering a unique phytochemical repertoire—tanshinones (lipophilic diterpenoids) and salvianolic acids (water-soluble phenolics)—that synergistically combat fibrosis through pleiotropic mechanisms. Preclinical studies highlight its capacity to suppress TGF-β1-mediated fibroblast activation and attenuate oxidative stress by enhancing Nrf2/HO-1 signaling (Li Q. et al., 2022), demonstrating its protective effects on cardiac health. The emphasis of TCM on syndrome differentiation and holistic approaches has garnered the attention of researchers worldwide. This article reviews related research on the use of *S. miltiorrhiza* in the treatment of MF.

## 2 Methodology

**FIGURE 1:**
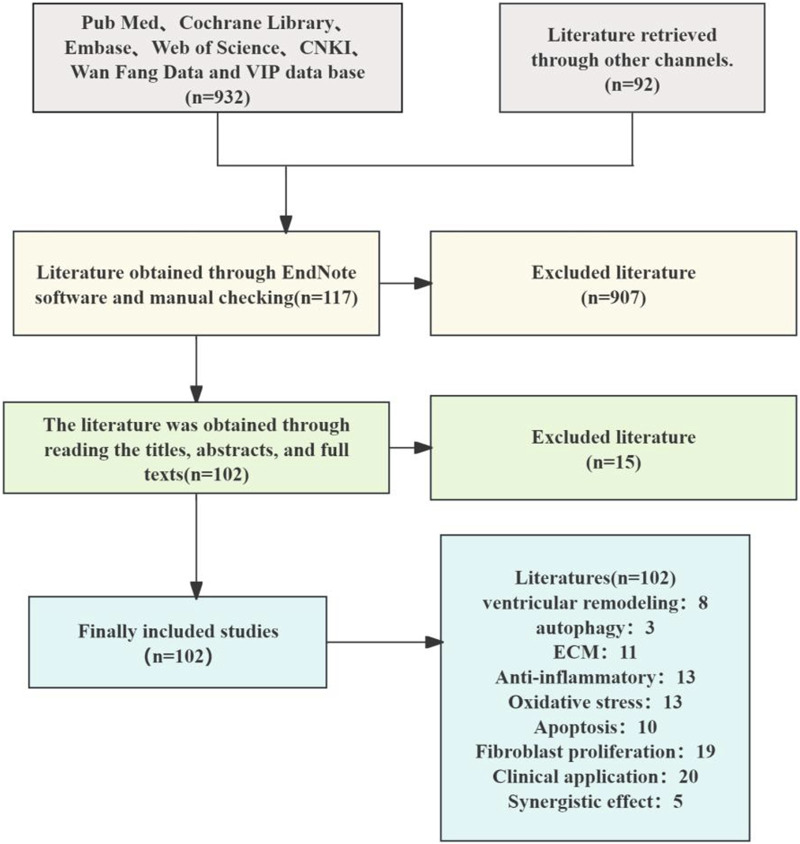
Literature screening flow chart.

This review article has been retrieved in the form of a database search. The search terms are in the form of subject words combined with free words. A systematic literature search was conducted across PubMed, Web of Science, CNKI, and Google Scholar. Search terms included “*Salvia miltiorrhiza* Bunge,” “active metabolites,” “pharmacological effects,” “extraction,” and “chemical structure.” (up to October 2024), total of 1,024 articles were retrieved, and 662 were duplicated by software and manual removal. After deduplication, 102 articles focused on *S. miltiorrhiza*’s anti-fibrotic mechanisms and clinical applications were retained. The research methods we included include clinical studies, clinical trials, cell experiments, animal experiments, literature reviews, network pharmacology, etc. We extracted study details, including the relevant information on the pharmacological action and chemistry attributes of *S. miltiorrhiza*, as well as the study status (Figure 1).

Taxonomic validation of plant species was performed using the Medicinal Plant Names Services (MPNS) and Plants of the World Online databases: [http://mpns.kew.org/mpns-portal/] (http://mpns.kew.org/mpns-portal/): and the Plants of the World Online database: [http://www.plantsoftheworldonline.org] (http://www.plantsoftheworldonline.org).

### 2.1 Mechanism of MF

MFis a pathological process characterized by excessive extracellular matrix (ECM) deposition and fibroblast activation, driven by ischemic, inflammatory, or metabolic insults. It represents a common pathological feature in the final stage of various cardiovascular diseases (López et al., 2021). Myofibroblasts are phenotypically regulated fibroblasts. The expression of α-smooth muscle actin (α-SMA) can identify differentiated myofibroblasts in damaged tissues and participate in the repair or fibrosis of damaged myocardial tissues. After cardiac injury, changes in the matrix environment, the induction and release of growth factors and cytokines, and an increase in mechanical pressure dynamically regulate the phenotype of fibroblasts (Kong et al., 2014). Fibroblasts secrete many ECM structural proteins, enzymes, growth factors, and cytokines, which in turn lead to excessive deposition of extracellular collagen (Travers et al., 2016). The number of fibroblasts increases significantly in diseases such as myocardial infarction (Willems et al., 1994), pressure-overloaded and volume-overloaded myocardium (Wang et al., 2003), aging heart (Obas and Vasan, 2018) and alcoholic cardiomyopathy (Fernández-Solà, 2020), indicating that myofibroblast transdifferentiation is a marker of MF. After TGF-β binds to TβRII on the cell surface, it can promote the phosphorylation of the cytoplasmic domain of TβRI and then transmit signals through Smad-dependent or Smad-independent pathways, promote the transdifferentiation of myofibroblasts and promote the deposition of the ECM in the cardiac interstitium (Xue et al., 2019). Experimental studies have also shown that reactive oxygen species (ROS) can activate TGF-β, thus promoting the deposition of the ECM in the cardiac interstitium (Zhu et al., 2020).

**FIGURE 2:**
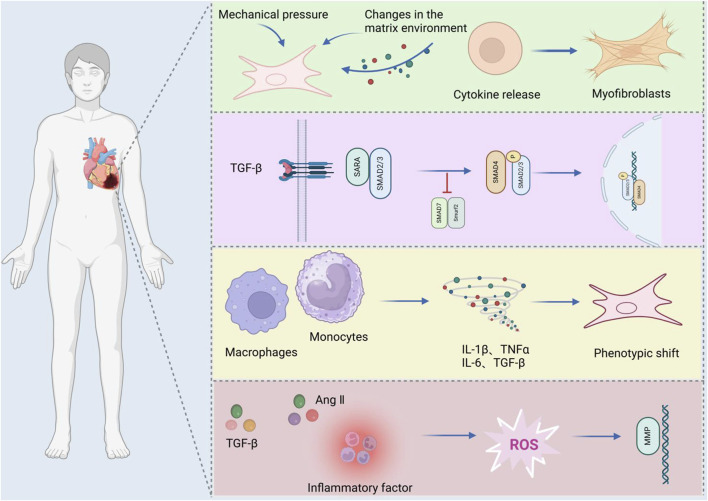
Mechanisms of MF.

ROS and angiotensin II (Ang II) synergistically promote fibroblast activation via redox-sensitive kinases (e.g., MAPK) and upregulation of pro-fibrotic genes. It can not only increase the transcription of MMPs by activating redox-sensitive kinases (Lijnen et al., 2012), but also mediate cardiac fibrosis and remodeling through Ang II-activated ROS-sensitive kinases. Cardiomyocyte apoptosis-related proteins are closely related to rat MF (Chiang et al., 2020). Specific monocyte and macrophage subsets play dual roles in fibrosis through their activity and microenvironment (Wynn and Barron, 2010), affecting fibroblast and matrix remodeling and inducing medium expression and release. These cells release proinflammatory mediators such as IL-1β, TNF-α, IL-6, TGF-β, and FGF to promote fibrosis. In fibrotic hearts, the expression of proinflammatory factors such as TNF-α, IL-1β, and IL-6 increases (Habib et al., 1996), which affects the fibroblast phenotype and gene expression (Siwik and Colucci, 2004), influences fibroblast and matrix remodeling, and induces medium expression and release (Timonen et al., 2008). In summary, MF pathogenesis involves crosstalk between TGF-β/Smad signaling, oxidative stress, inflammatory cascades (e.g., IL-6/STAT3), and apoptosis pathways (e.g., Bax/Bcl-2), as illustrated in Figure 2.

### 2.2 Overview of *Salvia miltiorrhiza* Bunge

**FIGURE 3:**
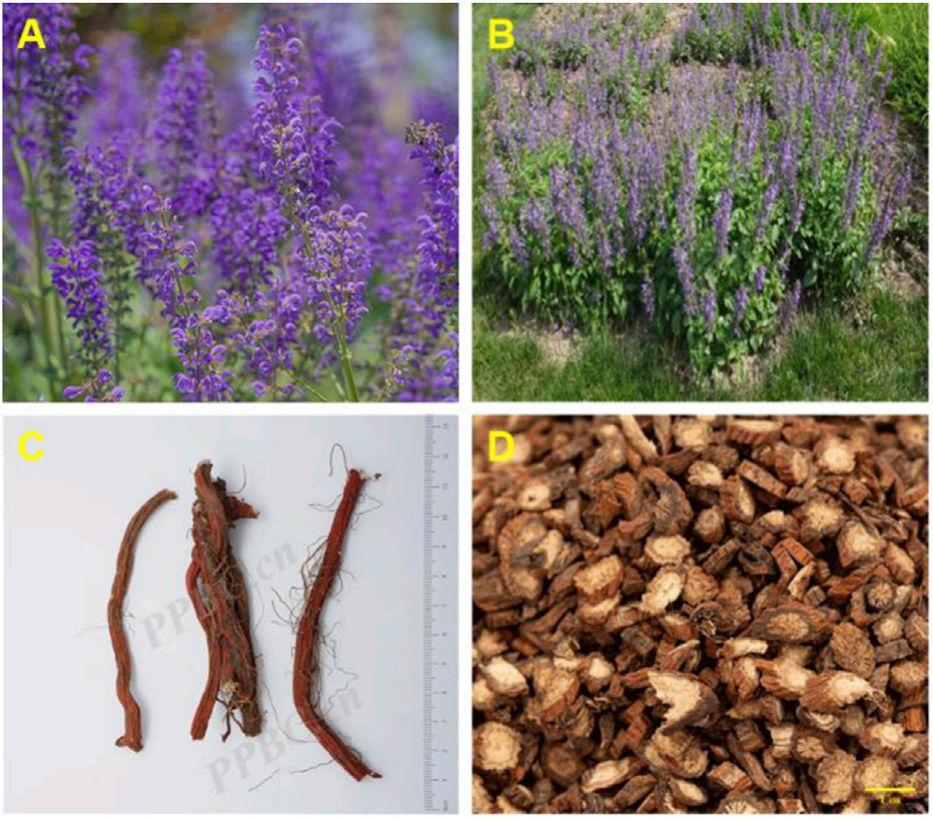
**(A, B)** *Salvia miltiorrhiza* botanical drugs (Plant Photo Bank of China, PPBC, http://ppbc.iplant.cn/), **(C)** *Salvia miltiorrhiza* original medicinal materialsand (Plant Photo Bank of China, PPBC, http://ppbc.iplant.cn/), **(D)** *Salvia miltiorrhiza* slices (Baidu library, https://xueshu.baidu.com/).

**FIGURE 4:**
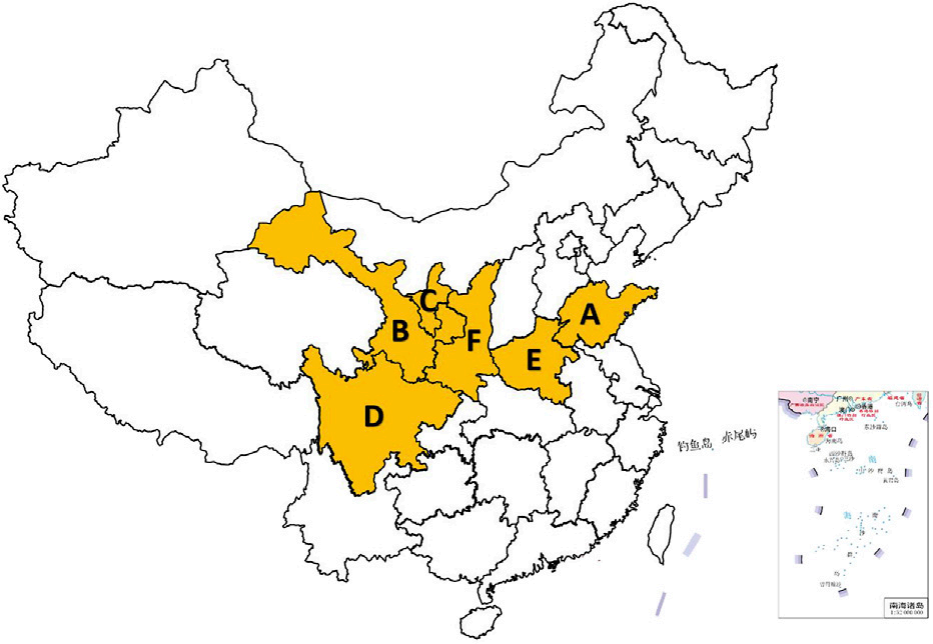
*Salvia miltiorrhiza* Bunge production areas in China. (A: Shandong, B: Gansu, C: ningxia, D: Sichuan, E: Henan, F: Shanxi) (The map of China is automatically generated by the WPS platform software. The distribution areas of *Salvia miltiorrhiza* Bunge are obtained based on records from literature and textbooks. These areas are highlighted in yellow to make the distribution more evident and easier for readers to understand).

*S. miltiorrhiza*, commonly known as Dahongpao or red root, is the dried root and rhizome of *S. miltiorrhiza*. It was included in the 2020 “Chinese Pharmacopoeia” and is among the most extensively used TCMs in China (Figures 3A, B). The rhizomes are dark brown, twisted (10–30 cm in length), with a rough exterior and aromatic pale-yellow interior (Figures 3C, D). *S. miltiorrhiza*, a widely used TCM in clinical practice, was initially documented in “Shen Nong’s botanical drugal Classic”. This botanical drug is known for its ability to enhance health and address a range of medical conditions. Its pharmacological properties span cardiovascular protection (antiplatelet aggregation, anti-fibrotic), anti-inflammatory, antioxidant, and antitumor effects (Lijnen et al., 2012), along with antitumor effects, induction of apoptosis, promotion of microcirculation, enhancement of hemorheology, improvement in lipid metabolism, and inhibition of atherosclerosis (Chiang et al., 2020). Contemporary pharmacological studies have revealed that the primary active metabolites of *S. miltiorrhiza* include tanshinones, salvianolic acids, volatile oils, polysaccharides, nitrogen-containing metabolites, and various other chemical entities (Li Z. M. et al., 2018). The primary production of this plant occurs in Shandong, China, where it is renowned for its high yield and superior quality. Other notable regions of production include Henan, Shanxi, Sichuan, and Gansu, among others (Figure 4).

## 3 *Salvia miltiorrhiza* Bunge active metabolites

**FIGURE 5:**
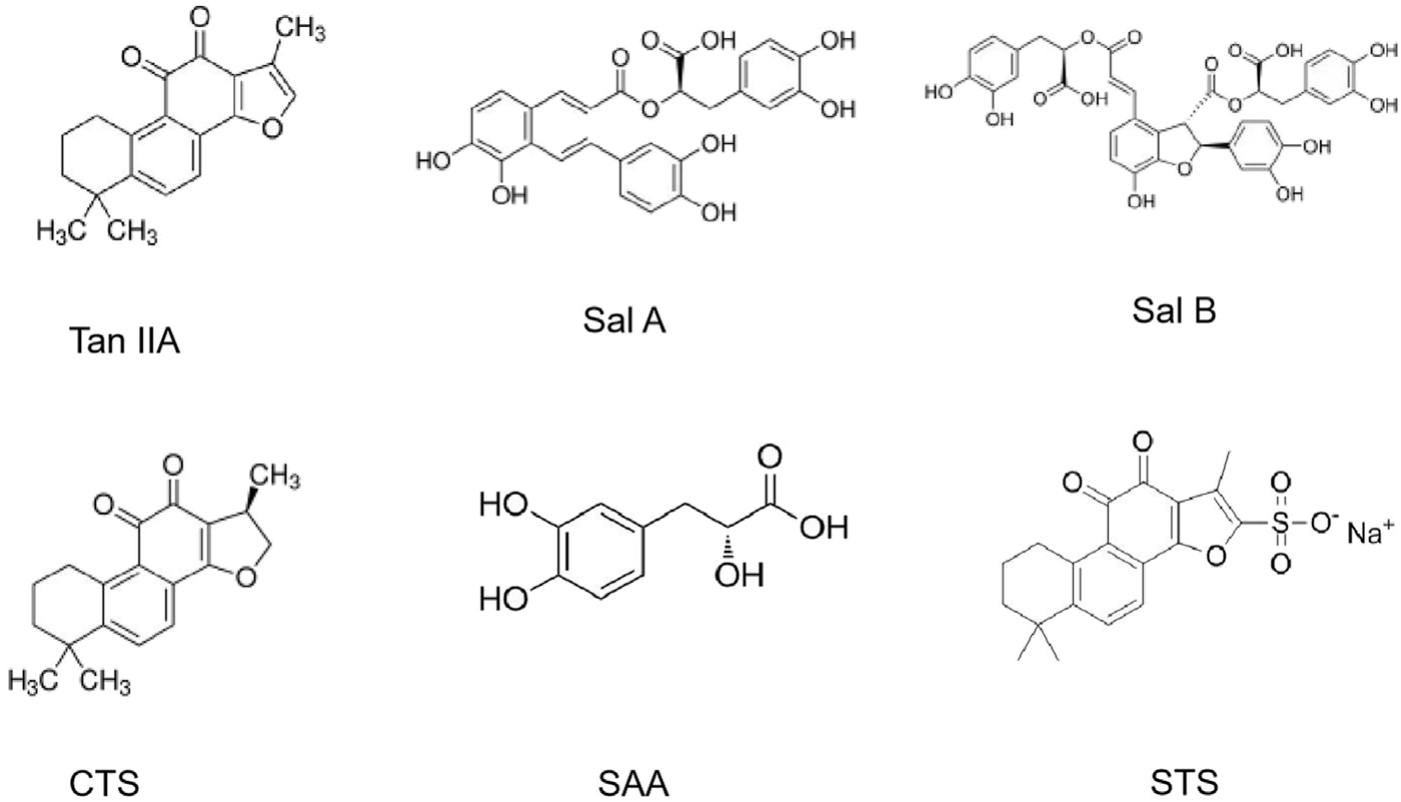
The chemical structures of the main active metabolites in *Salvia miltiorrhiza* Bunge.

The medicinal value of *S. miltiorrhiza* is derived from its complex chemical metabolites. More than 100 chemical metabolites have been isolated and identified. Over 100 bioactive metabolites are categorized into three classes: tanshinones (lipophilic diterpenoids), salvianolic acids (water-soluble phenolics), and volatile/polysaccharide derivatives. Ultra-fast liquid chromatography-mass spectrometry (UF-LC-MS) and thrombin inhibition assays identified salvianolic acids (e.g., salvianolic acid C) and tanshinones (e.g., cryptotanshinone) as potent thrombin inhibitors (Figure 5).

### 3.1 Tanshinones

The tanshinone metabolites in *S. miltiorrhiza* are mostly diterpenoids, which are fat soluble and are synthesized and accumulate in the periderm of *S. miltiorrhiza* roots. At present, more than 50 species have been isolated, such as tanshinone I, tanshinone IIA, tanshinone IIB, cryptotanshinone, dihydrotanshinone I, isocryptotanshinone, and tanshinones (Dong and Zheng, 2004). The biosynthetic pathway of tanshinone metabolites (Zeng et al., 2016) indicates that tanshinone diene is the first step in the formation of the skeletons of tanshinone metabolites. Through a series of biosynthetic pathways, tanshinone IIA and cryptotanshinone are ultimately formed under the catalysis of the cytochrome P450 enzymes oxidase, decarboxylase, dehydrogenase and reductase.

### 3.2 Salvianolic acids

Salvianolic acids in *S. miltiorrhiza* are water soluble and are synthesized and accumulate mainly in the phloem and xylem of its roots. At present, more than 30 kinds of salvianolic acids have been isolated. The main active metabolites are salvianolic acid A (Sal A), salvianolic acid B (Sal B), danshensu, caffeic acid, rosmarinic acid, protocatechuic aldehyde, lithospermic acid, etc. Most salvianolic acid metabolites can be regarded as derivatives of caffeic acid. For example, rosmarinic acid is a dimer of caffeic acid and danshensu, salvianolic acid B is a dimer of rosmarinic acid, and salvianolic acid A is formed by the condensation of one molecule of danshensu and two molecules of caffeic acid (Shi et al., 2019).

### 3.3 Other metabolites

The volatile oil content of Radix *S. miltiorrhiza* is low. At present, more than 30 metabolites have been isolated and identified from the volatile oil of Radix *S. miltiorrhiza*, including peach tocopherol, rust alcohol, caryophyllene, 7-isopropyl-1,1,4α-trimethyl-1,2,3,4,4α,9,10,10α-octahydrophenanthrene lactone, n-hexadecanoic acid, diisobutyl phthalate, germacrene D, oleic acid and n-eicosane (Li Y. et al., 2021). Polysaccharides in *S. miltiorrhiza* have attracted widespread attention because of their ability to enhance immunity and protect the liver (Chen Y. et al., 2017). Wang et al. (2014) isolated the polysaccharide SMPA from *S. miltiorrhiza*, which is composed of galactose, glucose, rhamnose, mannose and glucuronic acid. In addition, *S. miltiorrhiza* contains lactones such as salviamone and spiroketalide (Kong and Liu, 1983).

## 4 The mechanism of action of *Salvia miltiorrhiza* Bunge in the prevention and treatment of MF

### 4.1 Inhibition of ventricular remodeling

Ventricular remodeling refers to structural and functional changes in the myocardium in response to mechanical and nerve stimulation, including cardiomyocyte hypertrophy, extracellular matrix remodeling, and fibroblast activation. MF is not only the result of ventricular remodeling but also the pathological basis of ventricular remodeling (Geva and Bucholz, 2021). Excessive ECM deposition in MF increases myocardial stiffness, reduces compliance, and accelerates ventricular remodeling through biomechanical stress. Ventricular remodeling promotes the development of MF. In the process of ventricular remodeling, cardiomyocyte hypertrophy and extracellular matrix remodeling further activate fibroblasts and form a vicious cycle (Nagalingam et al., 2022; O’Meara and Zannad, 2023). TGF-β1, Ang II, and other factors play key roles in MF and ventricular remodeling. They jointly promote the progression of heart disease by activating fibroblasts and inducing cardiomyocyte hypertrophy. In conclusion, MF and ventricular remodeling promote each other in pathophysiological processes, and their molecular mechanisms are intertwined, which together lead to the deterioration of cardiac structure and function. Therefore, inhibiting ventricular remodeling can provide a new strategy for the treatment of MF. Studies have shown that the active metabolites of *S. miltiorrhiza* and its preparations can improve MF by inhibiting ventricular remodeling through a variety of mechanisms (Li X. et al., 2021).

#### 4.1.1 Effective active metabolites of *Salvia miltiorrhiza* Bunge

**FIGURE 6:**
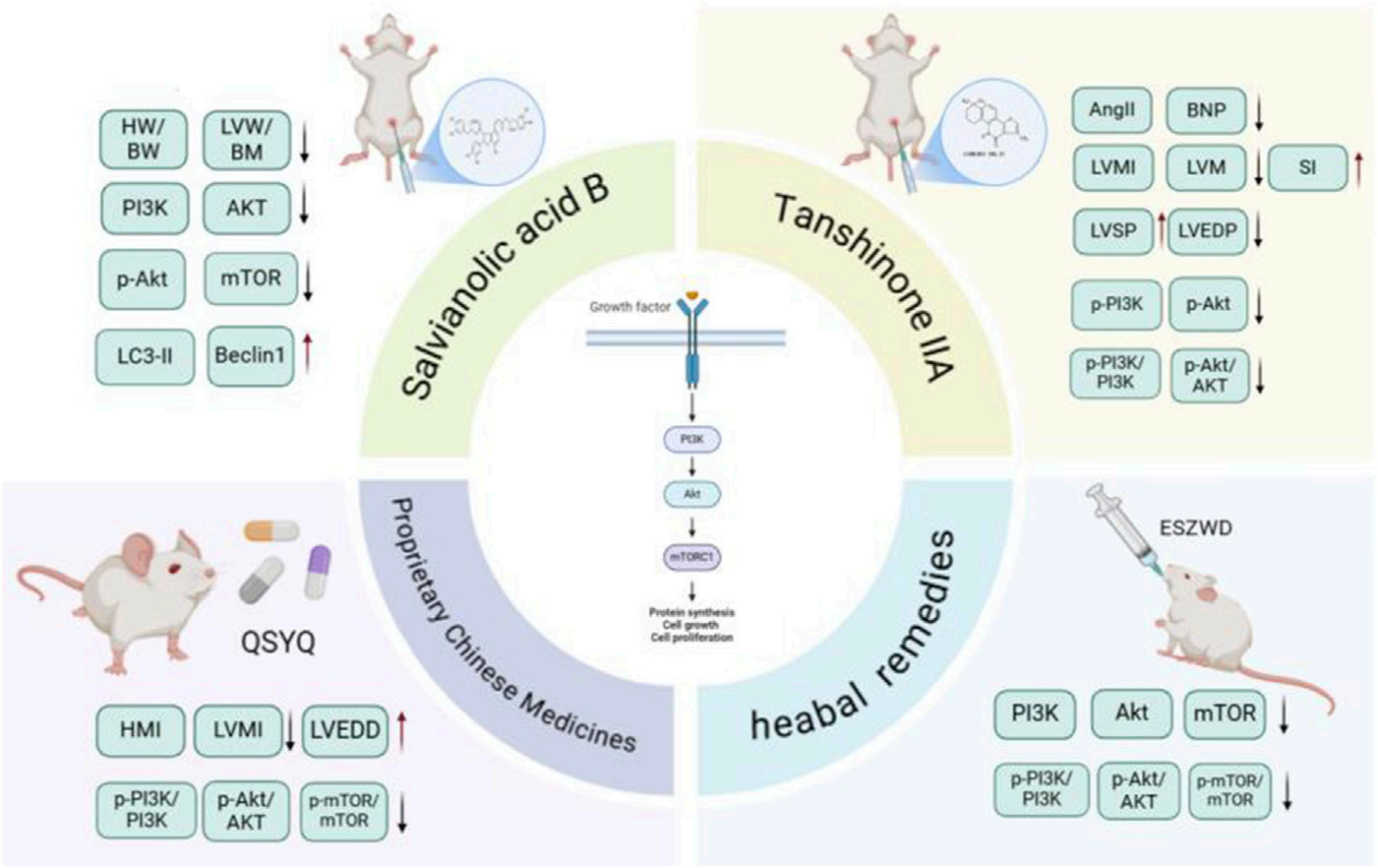
*Salvia miltiorrhiza* Bunge and its active metabolites regulate PI3K/Akt signaling pathway to prevent MF.

Tanshinone IIA (Tan IIA) is the main metabolite of *S. miltiorrhiza* and belongs to the class of diterpenoid quinones. Its molecular formula is C_19_H_18_O_3_, and its relative molecular weight is 294.33. It is obtained primarily through extraction, chromatography, crystallization, and other processes. Tan IIA exhibits cardioprotection via endothelial preservation, anti-arrhythmic effects, and attenuation of ischemia-reperfusion injury (Ansari et al., 2021). In a study conducted by Song et al. (2022) Tan IIA was administered at a dosage of 15 mg/kg via intraperitoneal injection for 28 consecutive days to rats with CHF induced by ISO. The results indicated that the Tan IIA group presented significantly lower levels of Ang II, brain natriuretic peptide (BNP), left ventricular mass index (LVMI), and left ventricular mass (LVM) than did the model group. Moreover, the Tan IIA group presented a lower degree of myocardial cell necrosis, MF, and remodeling. Notably, there was a reduction in the myocardial collagen volume ratio and protein levels of Col-I, Col-III, p-PI3K, and p-Akt in the myocardial tissue. He Dequan et al. (2022) established a rat model of myocardial hypertrophy via subcutaneous injection of ISO (5 mg/kg/d) for 14 consecutive days. Different doses (17.5 mg/kg/d, 35 mg/kg/d, and 70 mg/kg/d) of Tan IIA were used to treat the rats with MF. After 28 days of continuous intervention, the LVEDP, cardiac mass, cardiac index, COL-I, COL-III, p-PI3K, and p-AKT levels in the myocardial tissue of the Tan IIA intervention group and PI3K inhibitor group were significantly decreased, and the LVSP and ±dp/dtmax were significantly increased. These findings suggest that Tan IIA can reduce the phosphorylation levels of the PI3K and AKT proteins in the myocardial tissue of rats in a dose-dependent manner, improve ventricular remodeling and inhibit MF. Tan IIA dose-dependently suppressed PI3K/AKT phosphorylation (p-PI3K, p-AKT) in myocardial tissue, thereby improving ventricular remodeling and MF (Figure 6).

Sal B is a water-soluble phenolic metabolite extracted from the traditional Chinese medicine *S. miltiorrhiza*. Its molecular formula is C36H30O16, and its relative molecular weight is 718.62. It has many biological activities, such as anti-oxidative, anti-inflammatory, and anti-fibrotic effects. It has been widely studied and applied to the treatment of cardiovascular diseases (Liang et al., 2024). Gao et al. (2019) performed *in vivo* and *in vitro* studies and revealed that varying doses of Sal B (80 mg/kg/d and 160 mg/kg/d) significantly reduce the myocardial collagen area in ISO-induced MF in mice in a dose-dependent manner. Additionally, they reported a decrease in the protein expression levels of TGF-β1, Smad2, and Smad3, along with an increase in Smad7 expression. Sal B can regulate the TGF-β1/Smad signaling pathway in CFs to inhibit ventricular remodeling and improve MF. Li et al. (2020) established a type 1 diabetes model using streptozotocin (STZ) and reported that after 16 weeks of Sal B administration via intraperitoneal injection at doses of 15 mg/kg/d and 30 mg/kg/d, left ventricular dysfunction in diabetic mice improved considerably, and collagen deposition in the cardiac tissue decreased. Both *in vitro* and *in vivo* studies demonstrated that Sal B facilitated the phosphorylation of extracellular signal-regulated protein kinase and protein kinase B (AKT), thus promoting cellular proliferation. These findings indicate that Sal B may increase angiogenesis by inhibiting IGFBP3, which in turn mitigates MF and cardiac remodeling associated with diabetic cardiomyopathy.

Rosmarinic acid (RA) is a water-soluble phenolic acid that is synthesized via the condensation of Danshensu and caffeic acid. Studies have shown that it has antiviral, antibacterial, anti-inflammatory, and other effects (Guan et al., 2022). Zhang et al. (2018) induced ventricular remodeling via aortic ligation and administered RA (100 mg/kg/d) via gavage. The results showed that RA reduced ventricular remodeling and inhibited MF through AMPKα/Smad 3 signal transduction.

#### 4.1.2 *Salvia miltiorrhiza* Bunge metabolite and its preparations

The Qiliqiangxin capsule (QLQXC) is a polyherbal formulation composed of the following botanical drugs:*Astragalus membranaceus* (Fisch.) Bunge [Fabaceae; Astragali Radix], *Panax ginseng* C.A. Mey. [Araliaceae; Ginseng Radix et Rhizoma], *Aconitum carmichaelii* Debx. [Ranunculaceae; Aconiti Lateralis Praeparata], *S. miltiorrhiza*, *Descurainia sophia* (L.) Webb ex Prantl [Brassicaceae; Descurainiae Semen], *Alisma orientale* (Sam.) Juzep. [Alismataceae; Alismatis Rhizoma], *Polygonatum odoratum* (Mill.) Druce [Asparagaceae; Polygonati Odorati Rhizoma], *Cinnamomum cassia* Presl [Lauraceae; Cinnamomi Cortex], *Carthamus tinctorius* L. [Asteraceae; Carthami Flos], *Magnolia officinalis* Rehd. et Wils. [Magnoliaceae; Magnoliae Cortex], *Citrus reticulata* Blanco [Rutaceae; Citri Reticulatae Pericarpium]. This commercial Chinese polyherbal preparation (CCPP) represents a synthesis of TCM principles and contemporary scientific advancements. Several clinical studies have been conducted, leading to the recognition of its efficacy and research findings by experts and researchers around the world. Zhang et al. (2017) developed a CHF rat model through abdominal aortic ligation and administered different doses of QLQXC (0.25 g/kg/d, 0.5 g/kg/d, and 1 g/kg/d). The authors reported that QLQXC treatment decreased the serum levels of BNP, lLVMI, type I CVF, and type III CVF and the protein expression of TGF-β1 and Smad3, indicating that QLQXC improved myocardial hypertrophy and inhibited MF by inhibiting the TGF-β1/Smad3 signaling pathway.

Shenqi Jianxin Prescription (SQJXP) (Zhao, 2022) is a decoction-free formula granule composed of *A. membranaceus*, *P. ginseng* C.A. Meyer [Araliaceae; Ginseng Radix Rubra], *Atractylodes macrocephala* Koidz. [Asteraceae; Atractylodis Macrocephalae Rhizoma], *S. miltiorrhiza*, *Poria cocos* (Schw.) Wolf [Polyporaceae; Poria], *Epimedium brevicornu* Maxim. [Berberidaceae; Epimedii Folium], and *C. cassia* Presl [Lauraceae; Cinnamomi Ramulus]. According to the recommended dosage of prescription, the ratio of prescription is 20:15:10:15:10:15:15:15:6. A CHF model was constructed through coronary artery ligation. Following intragastric administration of SQJXP for 8 weeks at dosages of 3.7 mg/kg/d, 7.4 mg/kg/d, and 14.8 mg/kg/d, the cardiac function of the rats in each intervention group was significantly improved compared with that of the model group. Additionally, there was a reduction in the area of MF and a decrease in the mRNA levels of TGF-β1, Smad3, and Caspase-3 to different degrees. Similarly, the protein expression levels of TGF-β1, p-Smad3, and Caspase-3 also decreased to different degrees. The SQJXP improved ventricular remodeling and inhibited MF, and a dose of 14.8 mg/kg/d was the best.

**FIGURE 7:**
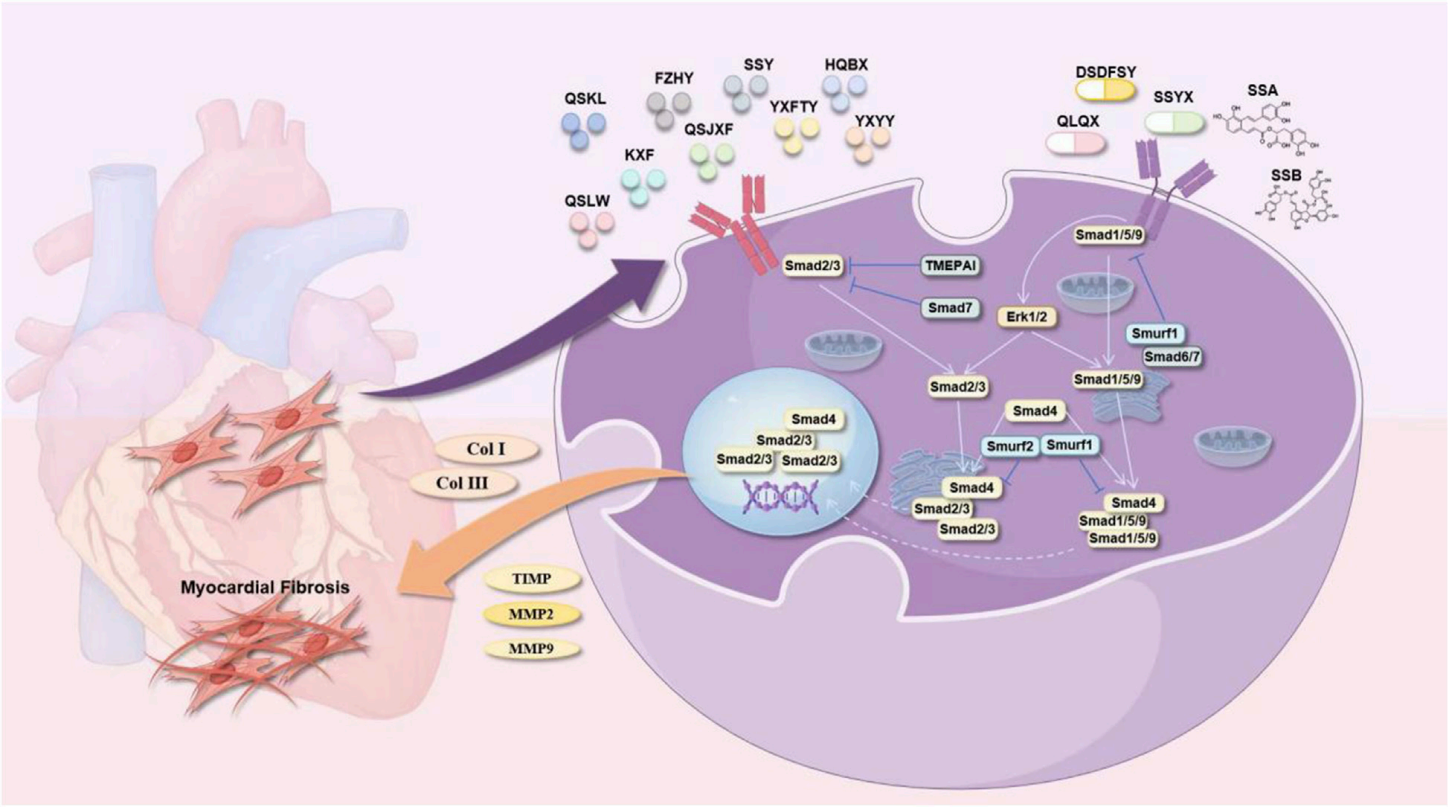
*Salvia miltiorrhiza* Bunge and its active metabolites regulate TGF-β/Smad signaling pathway to prevent MF.

Yixin Futing Yin (YXFTY) is a formula granule composed of the following botanical drugs: *A. carmichaelii*, *D. sophia A. macrocephala*, *C. cassia*, *Conioselinum anthriscoides* (Chuanxiong) [Apiaceae; Ligustici Chuanxiong Rhizoma], *P. cocos*, *Pseudostellaria heterophylla* (Miq.) Pax [Caryophyllaceae; Pseudostellariae Radix], *S. miltiorrhiza*, *Ophiopogon japonicus* (Thunb.) Ker Gawl. [Asparagaceae; Ophiopogonis Radix], *Rehmannia glutinosa* (Gaertn.) Libosch. ex DC. [Scrophulariaceae; Rehmanniae Radix], *Schisandra chinensis* (Turcz.) Baill. [Schisandraceae; Schisandrae Fructus], and *Glycyrrhiza glabra* L. [Fabaceae; Glycyrrhizae Radix et Rhizoma] formulated in the following proportions: 20:30:15:12:12:30:30:30:12:15:6:10. This formulation has pharmacological properties that help ameliorate myocardial hypertrophy. Liu et al. (2021) reported that administering 3.5 g/kg/d YXFTY significantly decreased collagen levels in rats with CHF. The treatment also led to a reduction in the percentage of fibrous tissue expression area and the expression levels of TGF-β1, Smad3, and Smad7 mRNA in myocardial tissue. These findings suggested that YXFTY may effectively inhibit excessive activation of the TGF-β/Smad signaling pathway, thus mitigating MF and ventricular remodeling in rats experiencing pressure-induced CHF (Figure 7). Table 1 summarizes *S. miltiorrhiza*’s anti-fibrotic mechanisms and clinical formulations targeting ventricular remodeling.

**TABLE 1:** Effective active metabolites and metabolite preparations of *Salvia miltiorrhiza* Bunge in inhibiting ventricular remodeling and improving MF.

Metabolite/metabolite	Experimental type	Model	Mechanism of action	Optimal dose	References
Tan IIA	Wistar rats	ISO	Reduced expression of p-PI3K and p-Akt proteins	15 mg/kg/d	Song et al. (2022)
Tan IIA	SD rats	ISO	Reduced expression of p-PI3K/PI3K and p-AKT/AKT proteins	70 mg/kg/d	He Dequan and Wang (2022)
Sal B	KM mice	ISO	Lower TGF-β1, Smad2, and Smad3, and boost Smad7	160 mg/kg/d	Gao et al. (2019)
Sal B	C57BL/6J mice	STZ/HUVEC	Regulate the PI3K/AKT pathway	30 mg/kg/d	Li et al. (2020)
RA	Male C57/B6 mice	LAD	Regulate the AMPKα/Smad 3 pathway	100 mg/kg/d	Zhang et al. (2018)
QLQXC	SD rats	AAC	Lower TGF-β1 and Smad3	1 g/kg/d	Zhang et al. (2017)
SQJXP	SD rats	LAD	Lower TGF-β1, Smad3, and Caspase-3 levels	14.8 mg/kg/d	Zhao (2022)
YXFTY	SD rats	AAC	Lower TGF-β1, Smad3, and Smad7 mRNA.	3.5 g/kg/d	Liu et al. (2021)

### 4.2 Regulating autophagy

Autophagy is a conserved intracellular degradation process in eukaryotes, essential for maintaining cellular homeostasis through lysosomal turnover of damaged components. Its essence is an intracellular metabolic process that responds to various external pressures. Damaged or misfolded proteins and organelles are sequestered by autophagosomes and then transferred into the lysosome for digestion and decomposition, thereby providing energy for cell metabolism and renewing the cell. Normal levels of autophagy are necessary for the human body to maintain the stability of the intracellular environment by promoting cell metabolism. Dysregulation occurs under pathological conditions such as nutrient deprivation or oxidative stress, the level of autophagy may be upregulated or downregulated. Excessive and insufficient autophagy may lead to disease (Li A. et al., 2022). In recent years, an increasing number of studies have shown that autophagy plays an important role in the occurrence and development of MF (Ambardekar and Sailer, 2022). Doxorubicin induces cardiac perivascular fibrosis via ROS-mediated NF-κB activation, which promotes endothelial-mesenchymal transition (EndMT) and autophagic dysfunction and cause cardiac toxicity. Irisin mitigates doxorubicin-induced cardiotoxicity by restoring UCP2-mediated autophagic flux and antioxidant defense, which confirms the protective effect of irisin on the microenvironment of cardiac microvascular endothelial cells and can be used as a potential therapeutic drug for doxorubicin-induced perivascular fibrosis. Many studies have shown that *S. miltiorrhiza* and its preparations have great potential in regulating autophagy in MF.

#### 4.2.1 Effectively active metabolites of *Salvia miltiorrhiza* Bunge

Du et al. (2019) demonstrated dose-dependent inhibition of ISO-induced MF by Sal B (15–30 mg/kg/d) in rats. Compared with those in the control group, the Sal B intervention groups presented decreases in the HW/BW, LVW/BW, and Col-I/Col-III ratios. Sal B treatment downregulated phosphorylated AKT/mTOR signaling while upregulating autophagy markers Beclin1 and LC3-II. H&E staining revealed that the degree of myocardial cell fibrosis in the Sal B intervention group was reduced, suggesting that Sal B can inhibit ISO-induced rat MF in a dose-dependent manner by inhibiting the PI3K/AKT/mTOR pathway to promote autophagy.

#### 4.2.2 *Salvia miltiorrhiza* Bunge metabolite and its preparations

Qishen Yiqi Dropping Pills (QSYQDP) are formulated from a combination of *A. membranaceus*, *S. miltiorrhiza*, *Panax notoginseng* (Burkill) F.H.Chen [Araliaceae; Notoginseng Radix et Rhizoma], and *Santalum album* L. [Santalaceae; Santali Albi Lignum]. In 2003, these pills were approved by the China Food and Drug Administration (CFDA) for their clinical application in the treatment of cardiovascular diseases. Lv et al. (2021) conducted animal experiments that demonstrated significant findings regarding the effects of varying doses of QSYQDP (135 mg/kg/d, 270 mg/kg/d, and 540 mg/kg/d) compared with a sham surgery group. This study revealed that QSYQDP administration led to a prominent reduction in HMI and LVMI, as well as a decrease in the myocardial collagen volume fraction. Additionally, QSYQDP treatment mitigated pathological alterations in myocardial tissue, resulting in the orderly and tightly organized arrangement of the MF. Studies have also revealed an increase in the number of myocardial autophagosomes, along with an increase in the expression levels of Beclin-1 and LC3-II/LC3-I in myocardial tissue and an inhibition of p62 expression. Additionally, the ratios of Akt, P-PI3K/PI3K, P-Akt/Akt, and P-mTOR/mTOR decreased in a dose-dependent manner, suggesting that QSYQDP activates myocardial autophagy via the PI3K/AKT/mTOR signaling pathway, thus exerting a dose-dependent anti-fibrotic effect on MF.

Er Shen Zhen Wu Decoction (ESZWD) is derived from the traditional formula Zhen Wu Decoction and is enhanced by the incorporation of two additional botanical drugal metabolites: *red ginseng* and *S. miltiorrhiza*. The primary metabolites of this formulation include *S. miltiorrhiza*, *Paeonia lactiflora* Pall. [Paeoniaceae; Paeoniae Radix Alba], *A. macrocephala.*, *P. cocos*, *P. ginseng*, and *A. carmichaelii* Debeaux [Ranunculaceae; Aconiti Lateralis Praeparata]; these metabolites are combined at a ratio of 30:10:10:10:6:6:5. Clinical studies have shown its effectiveness in the treatment of patients with CHF. *In vivo* and *in vitro* studies (Zhao, 2023; Zhao et al., 2023) demonstrated that relative to the control, different doses of ESZWD (3.96 g/kg/d, 7.92 g/kg/d, and 15.84 g/kg/d) significantly decreased the expression levels of myocardial α-SMA, Col-I, and Col-III mRNAs, as well as the proteins p-PI3K, p-AKT, and p-mTOR. These findings suggest that it can regulate the PI3K/AKT/mTOR signaling pathway to reduce collagen production, increase autophagy and reduce MF. Table 2 summarizes the autophagy-regulatory effects of *S. miltiorrhiza* and its bioactive components in MF models.

**TABLE 2:** Effective active metabolites and metabolite preparations of *Salvia miltiorrhiza* Bunge in regulating autophagy and improving MF.

Metabolite/metabolite	Experimental type	Model	Mechanism of action	Optimal dose	References
Sal B	SD rats	ISO	PI3K, AKT, p-AKT, and mTOR levels were reduced	30 mg/kg/d	Du et al. (2019)
QLQXDP	Wistar rats	AAC	The ratios of P-PI3K/PI3K, P-Akt/Akt, and P-mTOR/mTOR were decreased	540 mg/kg/d	Lv et al. (2021)
ESZWD	SD rats/CFs	Dox	Reduced PI3K, AKT, and mTOR expression in myocardial tissue and cells	15.84 g/kg/d	Zhao (2023), Zhao et al. (2023)

### 4.3 Effects on the degradation of ECM

The key characteristic of MF is the aberrant buildup of collagen fibers in the heart muscle, predominantly due to an imbalance in collagen synthesis and degradation. Collagen degradation is modulated by extracellular MMPs and tissue inhibitors of metalloproteinases (TIMPs). MMPs are zinc-dependent proteases critical for ECM remodeling, particularly in post-infarction cardiac remodeling and are significant factors in cardiac remodeling following myocardial infarction (Fan et al., 2014). Tissue inhibitor of metalloproteinase-1 (TIMP-1) is a glycoprotein found in various body fluids and tissues. It can inhibit the activity of nearly all MMPs, with particular efficacy against MMP-1, MMP-3, and MMP-9 (Wang et al., 2015). With respect to collagen degradation, TIMP-1 and MMPs play key roles in the preservation of normal myocardial architecture and functionality. Alterations in the TIMP-1/MMP ratio can lead to an imbalance between collagen synthesis and degradation, contributing to the development of MF. *S. miltiorrhiza* influences the degradation of the ECM by modulating the level of expression of MMPs and TIMPs, thus mitigating the progression of MF.

#### 4.3.1 Effective active metabolites of *Salvia miltiorrhiza* Bunge

Mao et al. (2014) reported that 0.1–10 mM Tan IIA downregulated Col-I collagen gene expression and collagen deposition in HCFs by regulating the PKA/CREB phosphorylation pathway while increasing the production of new elastic fibers. Tan IIA upregulated the synthesis of MMP-1 and downregulated the levels of MMP-2 and MMP-9. The results showed that Tan IIA interacts with non-canonical estrogen receptors to maintain an appropriate balance between the net deposition of collagen and elastin so that the newly deposited matrix has the best durability and elasticity.

Sodium Tan IIA sulfonate (STS) is a derivative of Tan IIA and is characterized as a water-soluble metabolite synthesized through the sulfonation of fat-soluble active metabolites derived from *S. miltiorrhiza*. Its chemical formula is C_19_H_17_O_3_·SO_3_Na, and its relative molecular weight is 396.39. This metabolite has various pharmacological properties, including a reduction in myocardial infarction size, a decrease in myocardial oxygen consumption, protection of myocardial cells, enhancement of myocardial contractility, amelioration of myocardial metabolic disorders, and inhibition of platelet aggregation. *In vitro* studies indicated that (Yang et al., 2009) it enhances the expression and activity of MMP-1 in CFs stimulated with Ang II while inhibiting myofibroblast differentiation.

Cryptotanshinone (CTS) is a diterpenoid quinone metabolite extracted from *S. miltiorrhiza*. It has a variety of biological activities, such as anti-inflammatory, antibacterial, antioxidant, anti-fibrotic (Zhang et al., 2019) and anti-tumor effects. Its chemical formula is C_19_H_20_O_3_, and its relative molecular weight is 296.36 (Li H. et al., 2021). CTS (10 mg/kg/d) reduced cardiac fibrosis in STZ-treated rats. In addition, the mRNA and protein levels of signal transducer and activator of transcription 3 (STAT 3), MMP-9 and connective tissue growth factor were decreased by CTS in DCM. *In vivo* and *in vitro* experiments have shown that CTS can inhibit MF by inhibiting the STAT 3 pathway in diabetic rats with MF (Lo et al., 2017). Another study revealed that (Ma S. et al., 2012) CTS (20 mg/kg/d) could upregulate MMP-2 in the myocardial tissue of ISO-induced MF mice. In addition, *in vitro* experiments revealed that CTS dose-dependently upregulated and activated MMP-2 in cultured CFs, indicating that the anti-MF effects of CTS regulate MMP-2.

#### 4.3.2 *Salvia miltiorrhiza* Bunge metabolite and its preparations

Lv et al. (2017) constructed a rat model of experimental autoimmune myocarditis via cardiac myosin and reported that QSYQDP effectively reduced HYP, PICP, and the PICP/PIIINP ratio in the myocardium. Compared with those in the model group, there was a significant increase in MMP-1 and tissue inhibitor of TIMP-1 mRNA, along with a decrease in the MMP-1/TIMP-1 ratio, which improved MF. Animal studies (Lv et al., 2015) have indicated that QSYQDP (135 mg/kg/d) significantly reduces HMI, LVMI, HYP, PICP, and PIIIN levels in rats with abdominal aortic constriction. It also decreases the PICP/PIIINP ratio and downregulates the expression of MMP-1 and TIMP-1 in myocardial tissue, inhibiting MF.

Qishen granule (QSG) is a modernized preparation of Zhenwu decoction, a classical Traditional Chinese Medicine formula. It is prepared from *A. membranaceus*, *S. miltiorrhiza*, *Lonicera japonica* Thunb. [Caprifoliaceae; Lonicerae Japonicae Flos], Scrophularia ningpoensis Hemsl. [Scrophulariaceae; Scrophulariae Ningpoensis Radix], *Cyperus rotundus* L. [Cyperaceae; Cyperi Rhizoma], and *G. glabra* at a ratio of 30:15:10:10:9:6. Tan et al. (2022) reported that QSG (1 mg/mL) downregulates MMP-2, MMP-9, TIMP-1, and TIMP-2 in Ang II-stimulated CFs. It also decreases the MMP-2/TIMP-2 and MMP-9/TIMP-1 ratios, inhibits fibroblast proliferation, downregulates the expression of Col-I and Col-III, and modulates ECM metabolism.

Qi Shen Liu Wei formula granules (QSLWFGs) were prepared from *A. membranaceus*, *S. miltiorrhiza*, *C. anthriscoides*, *Pueraria montana* var. lobata (Willd.) Maesen & S.M.Almeida ex Sanjappa & Predeep [Fabaceae; Puerariae Lobatae Radix], *R. glutinosa* (Gaertn.) Libosch. ex DC. [Scrophulariaceae; Rehmanniae Radix], *A. orientale*, *Leonurus japonicus* Houtt. [Lamiaceae; Leonuri Herba], *P. notoginseng*, *Cornus officinalis* Siebold & Zucc. [Cornaceae; Corni Fructus], and *Prunella vulgaris* L. [Lamiaceae; Prunellae Spica] were mixed at ratios of 30:30:30:30:15:15:15:10:10:10:10. An animal study (Hui et al., 2023) revealed that QSLWFG (34.16 g/kg/d) can decrease the percentage of collagen fiber area in myocardial tissue and the protein expression of Col I, Col III, MMP-9, TGF-β1, Smad2, and Smad3 by regulating the TGF-β1/Smad2/3 signaling pathway. This helps alleviate the deposition of the ECM and improves MF in SHRs.

Fuzheng Huayu capsule (FZHYC), made from *S. miltiorrhiza*, *Juglans regia* L. [Juglandaceae; Juglandis Semen], *Pinus massoniana* Lamb. [Pinaceae; Pinus Massoniana Pollen], *S. chinensis*, *Gynostemma pentaphyllum* (Thunb.) Makino [Cucurbitaceae; Gynostemmatis Pentaphylli Herba], and *Cordyceps mycelium* [Ophiocordycipitaceae; Cordyceps Mycelium], is a CCPP for fibrosis. It alleviates hepatic fibrosis and is effective against renal and pulmonary fibrosis (Liu et al., 2005). Qi Yifei and colleagues (Qi et al., 2018) demonstrated that 0.4 g/kg FZHYC effectively inhibited MF in rat models by increasing the expression of the miRNA-29 family, which balances MMPs and TIMPs, specifically MMP2/TIMP2 and MMP9/TIMP1, enhancing ECM metabolism and reducing collagen deposition.

Fuzheng Huayu Prescription (FZHYP) is composed of *S. miltiorrhiza*, *C. mycelium*, *J. regia*, *G. pentaphyllum*, *P. massoniana*, and *S. chinensis* at a ratio of 8:4:2:6:2:2. Zhu et al. (2019b) reported that FZHYP (25 μg/mL, 50 μg/mL, and 100 μg/mL) can reduce the content of Col-I and Col-III and the expression of MMP2, MMP9, TIMP1 and TIMP2 mRNAs and downregulate the expression of the TGF-β1, p-Smad2, Smad3, and p-Smad3 proteins in Ang II-induced CFs compared with those in the control group. Therefore, the cellular and molecular mechanism of its anti-myocardial fibrosis effects may be related to the regulation of TGF-β/Smad signaling pathway-mediated matrix metabolism via the targeting of miR-29b-5p.

Yiqi Huoxue metabolite (YQHXC) is composed of *A. membranaceus*, *P. ginseng*, *C. tinctorius*, *S. miltiorrhiza*, *P. notoginseng*, and *D. sophia* effectively reduces ventricular remodeling *in vivo*. This effect is achieved by inhibiting the expression of MMP-1 and increasing Col-III levels while decreasing MF when combined with exercise (Li et al., 2011). Table 3 summarizes *S. miltiorrhiza*’s regulatory effects on ECM degradation enzymes in myocardial fibrosis models.

**TABLE 3:** Effective active metabolites and metabolite preparations of *Salvia miltiorrhiza* Bunge in effects on the degradation of the ECM and improving MF.

Metabolite/metabolite	Experimental type	Model	Mechanism of action	Optimal dose	References
Tan IIA	HCF	—	Regulating the PKA/CREB pathway boosts MMP-1 synthesis but reduces MMP-2 and MMP-9 levels	10 μM	Mao et al. (2014)
STS	CFs	Ang II	Boost MMP-1expression and activity	30 μM	Yang et al. (2009)
CTS	Wistar rat	STZ	Inhibition of STAT 3 pathway	10 mg/kg/d	Lo et al. (2017)
CTS	C57 BL/6 mice	ISO	Regulate MMP-2	20 mg/kg/d	Ma et al. (2012a)
QSYQDP	Lewis rats	myocardial myosin	MMP-1 and TIMP-1 mRNA are upregulated, but the MMP-1/TIMP-1 ratio is reduced	135 mg/kg/d	Lv et al. (2017)
QSYQDP	Wistar rats	LAC	downregulates the expression of MMP-1 and TIMP-1	135 mg/kg/d	Lv et al. (2015)
QSG	CFs	AngⅡ	reduce MMP-2/TIMP-2 and MMP-9/TIMP-1 ratios	1 mg/mL	Tan et al. (2022)
QSLWFG	SHR/WKY rats	—	Regulate the TGF-β1/Smad2/3 signaling pathway and inhibit ECM degradation	34.16 g/kg/d	Hui et al. (2023)
FZHYC	SD rats	LAD	Reduced Collagen 1, Collagen 3, and mRNA expression, as well as decreased MMP-2/9, TIMP-1/2, and their mRNA levels	0.4 g/kg/d	Qi et al. (2018)
FZHYP	CFs	Ang II	reduce the ratios of MMP-2/TIMP-2 and MMP-9/TIMP-1	100 μg/mL	Zhu et al. (2019b)
YQHXC	SD rats	LAD	MMP-1 mRNA and protein expression are downregulated	9.2 g/kg/d	Li et al. (2011)

### 4.4 Anti-inflammatory effects

The immune-inflammatory response is central to the pathogenesis of MF, mediated by key inflammatory factors including TNF-α, IFN-γ, IL-6, IL-1β, CRP, MCP-1, and ICAM-1 Inflammatory mediators increase fibroblast expression, alter the myocardial interstitial composition, and promote fibroblast migration. ROS also induce MF through various mechanisms. Increased inflammatory cell activity leads to fibroblast proliferation and differentiation into myofibroblasts, resulting in greater collagen deposition and MF development (Prabhu and Frangogiannis, 2016). The NF-κB transcription factor, which is found mainly in cardiomyocytes, is involved in immune development, response, inflammation, and cancer. It regulates inflammatory responses and immune homeostasis and plays crucial roles in myocardial inflammation, apoptosis, and cardiac remodeling (Hong et al., 2020). The NF-κB signaling pathway mediates fibrotic diseases (Deng et al., 2023). Its activation increases the levels of proinflammatory cytokines, such as IL-1, IL-18, TNF-α, and iNOS (Xu et al., 2020). The activation of iNOS results in significant NO release, worsening tissue injury and contributing to MF and impaired cardiac systolic function (Peng et al., 2022). Tanshinone metabolites from *S. miltiorrhiza* have anti-inflammatory properties that inhibit inflammatory cytokines and reduce MF.

#### 4.4.1 Effectively active metabolites of *Salvia miltiorrhiza* Bunge

In a rat model of MF induced by abdominal aortic coarctation, Tan IIA (20 mg/kg/d) significantly downregulated hydroxyproline (HYP) and NF-κB p65 protein expression (Cai et al., 2014).

Luo et al. (2023) established a diabetic cardiomyopathy (DCM) mouse model through high-fat/high-sugar diet and intraperitoneal STZ injection. After the model was successfully established, different doses of Sal B (1.5 mg/kg/d, 3 mg/kg/d) were administered intragastrically. Sal B inhibited inflammatory cell infiltration, inhibited the TGF-β1 signaling pathway by upregulating Smad7, significantly improved cardiac function in DCM rats, inhibited collagen deposition and phenotypic transformation, and reduced MF. *In vitro* experiments revealed that Sal B significantly inhibited the proliferation, migration, phenotypic transformation, and collagen secretion of CFs induced by high glucose. *In vivo* and *in vitro* experiments have shown that Sal B may improve MF by deubiquitinating Smad7, stabilizing Smad7 protein expression, blocking the TGF-β1 signaling pathway, and inhibiting inflammatory cell infiltration.

#### 4.4.2 *Salvia miltiorrhiza* Bunge extract

Danhong injection (DHI) is a standardized extract from *S. miltiorrhiza* and *C. tinctorius* (Guan et al., 2013). This formulation has long been used in the clinic for treating ischemic encephalopathy and cardiovascular conditions, such as myocardial infarction and angina pectoris. Studies have revealed that the primary metabolites of DHI include the following metabolites: danshensu, hydroxysafflor yellow A, 5-hydroxymethyl-2-furfural, protocatechuic aldehyde, viologen acid, caffeic acid, Sal A, Sal B, Sal C, protocatechuic acid, and rosmarinic acid (Liu et al., 2013). Chen J. et al. (2019) reported that DHI administration in MI model rats reduces the serum levels of the inflammatory cytokines TNF-α, IL-1β, and IL-6. Interference also inhibits the phosphorylation of NF-κB and IκB-α, enhancing cardiac function and hemodynamic parameters.

#### 4.4.3 *Salvia miltiorrhiza* Bunge metabolite and its preparations

Studies (Ye et al., 2023) have shown that the use of the QLQXC can improve cardiac function, increase the 6-min walk distance (6 mwd), and increase the E/A ratio in individuals with HFpEF. Hao et al. (2023) reported that administering different doses of QLQXC (0.25 g/kg/d and 1 g/kg/d) over 8 weeks significantly reduced NF-κB, TGF-β1, MMP2, MMP9, Smad2, and Smad protein levels in the myocardial tissue of rats with heart failure with preserved ejection fraction (HFpEF). This treatment also lowered the serum TNF-α and IL-2 levels, improved diastolic dysfunction, prevented left ventricular hypertrophy, enhanced the inflammatory response, and improved myocardial function in HFpEF rats. Sustained administration of QLQXC (1.0 g/kg/d) (Han et al., 2018) for 4 weeks significantly reduces the serum TNF-α and IL-6 levels in rats with myocardial infarction. It also alleviates MF by decreasing α-SMA in myocardial tissue, inhibiting collagen synthesis, and suppressing CFs activation and myofibroblast formation. This effect is associated with the inhibition of the TGF-β1/Smad3 and NF-κB signaling pathways. Yingdong Lu and colleagues (Lu Y. et al., 2022) reported that QLQXC (100 mg/kg/d) improved myocardial cell organization in rats with CHF resulting from transverse aortic constriction. This is achieved by modulating the intestinal microbiota and the NLRP3 inflammasome, which decreases inflammatory infiltration. QLQXC treatment also decreases the expression of proinflammatory proteins such as IL-1β, NF-κB, and TNF-α in myocardial tissue, leading to improvements in ventricular remodeling, enhanced cardiac function, and MF.

Another study (Zhang et al., 2022b) reported that various doses of the metabolite Zhenzhu tiaozhi capsule (CFZZTZC) (1.2 g/kg/d and 2.4 g/kg/d) can downregulate mRNA expression, inhibit cardiac inflammation, and improve myocardial function in mice with pressure overload.

The Zuo Gui Jiang Tang Shu Xin Prescription (ZGJTP) is composed of *P. ginseng*, *A. membranaceus*, *O. japonicus*, *C. officinalis*, *R. glutinosa*, *Coptis chinensis* Franch. [Ranunculaceae; Coptidis Rhizoma], *S. miltiorrhiza*, *P. montana* var. *lobata*, and *Crataegus monogyna* Jacq. [Rosaceae; Crataegi Fructus] at a ratio of 18:18:12:12:15:6:9:12:9. It can promote the apoptosis of myocardial cells and inhibit damage to myocardial cells. Huang et al. (2024) reported that ZGJTP (16.84 g/kg/d and 33.67 g/kg/d) effectively reduced the serum levels of TNF-α and IL-1β in MKR mice with diabetic cardiomyopathy. Furthermore, the treatment significantly downregulated the protein and mRNA expression levels of Col-I, Col-III, α-SMA, TLR4, and NF-κB p56 in myocardial tissues while also suppressing the phosphorylation of NF-κB p56. Mechanistically, this study demonstrated that ZGJTP exerts anti-MF effects through the modulation of the TLR4/NF-κB signaling pathway, thereby inhibiting inflammatory factor production.

**FIGURE 8:**
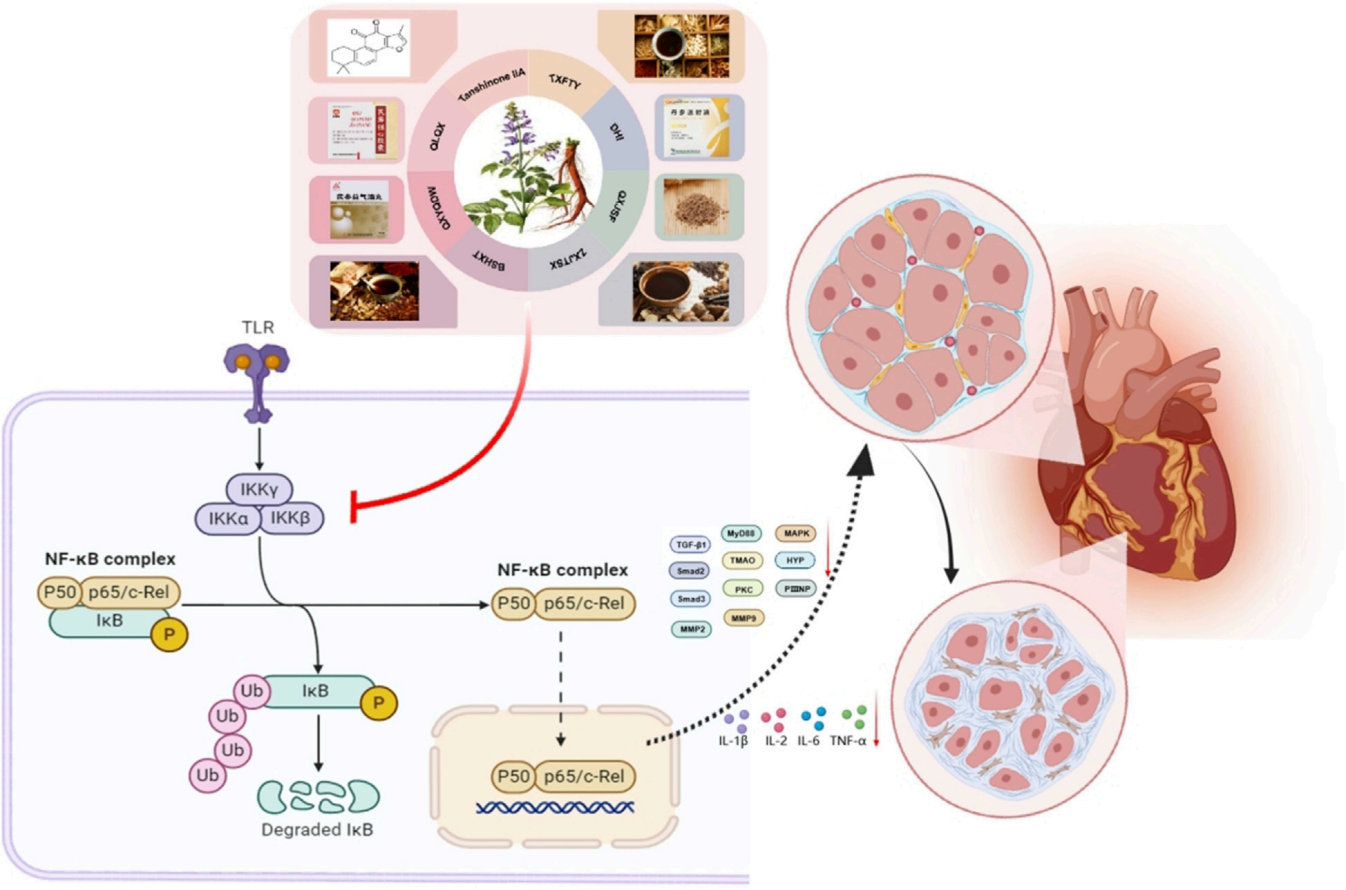
*Salvia miltiorrhiza Bunge* and its active metabolites regulate NF-κB signaling pathway to prevent MF.

Yangxin Tongmai Prescription (YXTMP) consists of equal parts of *P. ginseng*, *S. miltiorrhiza*, *C. cassia*, *Citrus × aurantium* f. aurantium [Rutaceae; Aurantii Fructus Immaturus], and *A. orientale*. It alleviates intestinal barrier dysfunction, regulates the intestinal flora, reduces inflammatory cytokines, inhibits ventricular remodeling, and enhances cardiac function. YXTMP (12 g/kg/d) effectively reduces (Xiao et al., 2023) the serum TNF-α, IL-1β, and IL-6 levels while increasing the IL-10 level in CHF rats after 4 weeks. Masson staining revealed a decrease in MF and an improvement in cardiac function (Figure 8).

The Qi Shen Yi Qi Prescription (QSYQP) consists of *A. membranaceus*, *S. miltiorrhiza*, *C. anthriscoides*, and *P. ginseng* at a ratio of 15:12:12:3. A previous study (Zhao, 2016) revealed that QSYQP (0.7 g/kg/d and 1.4 g/kg/d) reduces serum IL-1β, IL-6, and TNF-α levels, inhibiting MF in hypertensive murine models.

YXFTY (3.5 g/kg/d) alleviates myocardial fibrosis in heart failure (HF) rats by suppressing collagen I/III deposition and NF-κB p65 protein expression (Zhang et al., 2022c).

Bushen Huoxue Decoction (BSHXD) is a combination of *P. odoratum*, *S. miltiorrhiza*, *Cuscuta chinensis* Lam. [Convolvulaceae; Semen Cuscutae], *A. membranaceus*, *G. glabra*, *P. ginseng*, *L. japonicus*, and *P. notoginseng*, with the respective ratios of these metabolites being 20:30:20:40:10:15:20:5. Xu R. et al. (2024) reported that BSHXD at 1.575 g/mL modulates key proteins in the p38MAPK/p65NF-κB/AQP4 signaling pathway, influences the intestinal microbiota and metabolites, enhances intestinal barrier function, mitigates cardiomyocyte hypertrophy and fibrosis, and improves cardiac functions.

Yixintai (YXT) is a TCM formulation frequently used in the clinical management of HF. Its primary metabolites are composed of *A. membranaceus*, *S. miltiorrhiza*, *C. tinctorius*, *P. ginseng*, *A. orientale*, *P. cocos*, *D. sophia*, and *P. cocos*, along with other medicinal substances. The ratio of the individual metabolites is as follows: 15:15:30:10:10:15:15:15. Studies have shown that YXT may enhance cardiac function and lower serum BNP levels in rat models of CHF (Shi et al., 2024). This study revealed that different doses of YXT (Wang Z. et al., 2024) (1.4 g/kg/d, 2.8 g/kg/d, and 5.6 g/kg/d) influenced cardiac function in rats with HF induced by left anterior descending artery ligation. Treatment decreased the expression of inflammatory markers (IL-1β, IL-6, and TNF-α), inhibited NF-κB and PKC expression, modulated the TMAO/PKC/NF-κB pathway, and decreased myocardial hypertrophy and fibrosis. The role of *S. miltiorrhiza* in regulating inflammation is shown in Table 4.

**TABLE 4:** Effective active metabolites and metabolite preparations of *Salvia miltiorrhiza* Bunge in inhibiting inflammation and improving MF.

Metabolite/metabolite	Experimental type	Model	Mechanism of action	Optimal dose	References
Tan IIA	SD rats	AAC	The levels of HYP and NF-κB p65 protein are decreased	20 mg/kg/d	Cai et al. (2014)
Sal B	C57BL/6J mice	STZ	inhibit TGF-β1 signaling pathway by up-regulating Smad 7	3 mg/kg/d	Luo et al. (2023)
DHI	SD rats	LAD	Downregulation of serum TNF-α, IL-1β, IL-6 and myocardial tissue NF-κB and IκB-α protein expression	—	Chen et al. (2019a)
QLQXC	SD rats	HFpEF	The levels of TGF-β1, MMP2, MMP9, Smad2, Smad3, and NF-κB proteins were reduced	1 g/kg/d	Hao et al. (2023)
QLQXC	SD rats	LAD	Serum TNF-α and IL-6 levels decreased along with myocardial α-SMA and NF-κB p63 content	1 g/kg/d	Han et al. (2018)
QLQXC	SD rats	Transverse aortic coarctation	The expression of IL-1β, NF-κB and TNF-α protein in myocardial tissue was downregulated	100 mg/kg/d	Lu et al. (2022b)
CFZZTZC	C57BL/6/CFs	transverse aortic constriction/Ang II	The expressions of mRNA and proteins for HYP, Col1A2, Col3, CTGF, IL-1β, MCP-1, and TNF-α in myocardial tissue exhibited a downregulation	2.4 g/kg/d	Zhang et al. (2022b)
ZGJTP	MKR mice	STZ + high fat diet feeding	Suppressed protein expression of Col-I, Col-III, α-SMA, TLR4, and NF-κB p56	33.67 g/kg/d	Huang et al. (2024)
YXTMP	SD rats	LAD	The levels of TNF-α, IL-1β and IL-6 in serum were downregulated, and IL-10 was upregulated	12 g/kg/d	Xiao et al. (2023)
QSYQP	C57 mice	Ang II	The levels of serum IL-1β, IL-6, and TNF-α were found to be decreased	1.4 g/kg/d	Zhao (2016)
YXFTY	SD rats	Abdominal aortic banding	Reduce the area of myocardial fibers and downregulate the expression of Collagen 1, Collagen 3 and NF-κB p65 protein	3.5 g/kg/d	Zhang et al. (2022c)
BSHXD	SD rats	LAD + Exhaustive swimming + hunger method	It can regulate p38MAPK/p65NF-κB/AQP4 signaling pathway and improve MF	1.575 g/mL	Xu et al. (2024b)
YXT	SD rats	LAD	It can reduce the levels of serum inflammatory factors such as IL-1β, IL-6 and TNF-α, and regulate the TMAO/PKC/NF-κB signaling pathway	5.6 g/kg/d	Wang et al. (2024b)

### 4.5 Inhibiting oxidative damage

Oxidative stress arises from dysregulation between ROS generation and antioxidant capacity, contributing to cellular damage in MF. This can lead to tissue damage and is associated with many diseases. Oxidative stress generates reactive species such as ROS and reactive nitrogen species (RNS) In pathological states, an overproduction of oxygen free radicals or a weakened antioxidant defense can disrupt the balance between their generation and elimination, resulting in ROS accumulation. The onset and progression of MF are associated with oxidative stress (Kura et al., 2020). Oxidative stress activates NF-κB, leading to the production of TNF-α (Nizamutdinova et al., 2013). In myocardial injury, NF-κB translocates to the nucleus, binds to TNF-α, and initiates its transcription, contributing to MF. TNF-α also induces proto-oncogenes such as c-myc and c-fos, promoting fibrosis. A study (Li et al., 2000) revealed that oxygen free radicals enhance AT1R expression and mRNA via ox-LDL. They also increase Ang II synthesis by stimulating the release of endothelin, promoting VSMC proliferation and contributing to myocardial hypertrophic fibrosis.

#### 4.5.1 Effectively active metabolites of *Salvia miltiorrhiza* Bunge

Salvianic acid A (SAA), a phenolic aromatic acid from *S. miltiorrhiza*, is known as β-(3,4-dihydroxyphenyl) lactic acid. It has a white, needle-like crystalline structure and a molecular formula of C_9_H_10_O_5_. This study demonstrated that SAA (160 mg/kg) reduced hydroxyproline content and superoxide dismutase (SOD) activity while increasing malondialdehyde (MDA) levels in a LAD coronary artery occlusion-induced myocardial infarction mouse model. Additionally, it decreased the myocardial collagen volume fraction and SEC61α protein expression, suppressed oxidative stress, and ameliorated MF (Liu and Wang, 2021).

Yuan et al. (2019) reported that 15 mg/kg Tan IIA reduced myocardial oxidative stress and Nox4 expression in a rat model of heart failure. This treatment also increased SOD activity, alleviating MF. An animal study (Cai et al., 2016) revealed that 20 mg/kg Tan IIA enhances cytoprotection by increasing HO-1 expression, reducing MF, and delaying ventricular remodeling. Ruijuan Chen et al. (2021) reported that 1.5 mg/kg/d Tan IIA downregulated the mRNA levels of CoL-I, CoL-III, TGF-β, and α-SMA in a rat model of myocardial infarction. It also increased SOD activity and inhibited MF.

STS is a water-soluble derivative synthesized by sulfonation of Tan IIA extracted from *S. miltiorrhiza*. It has many pharmacological effects and is mainly used to treat cardiovascular diseases. Li Z. et al. (2018) reported that STS at doses of 5 mL/kg/d, 10 mL/kg/d, and 20 mL/kg reduced Ang II-induced MF in a dose-dependent manner. This treatment increased nuclear Nrf2 accumulation, increased the expression of antioxidant enzymes, improved the antioxidant response, and decreased lipid peroxidation in myocardial tissue. Yang et al. (2009) reported that 3 μM, 10 μM, and 30 µM STS effectively suppressed Ang II-induced CoL-I expression and inhibited ROS production while also modulating collagen and MMP-1 expression in CFs.

CTS is a bioactive metabolite extracted from *S. miltiorrhiza* that has multiple protective effects on cardiovascular diseases (Ma et al., 2014) CTS (30 mg/kg/d, 60 mg/kg/d) attenuated Ang II-induced upregulation of fibronectin, connective tissue growth factor, and cyclooxygenase-2 and normalized Ang II-induced upregulation of extracellular signal-regulated kinase 1/2 (ERK 1/2). Moreover, CTS inhibited Ang II-stimulated upregulation of NAD(P) H oxidase 2 and 4 (NOX-2 and NOX-4) and reactive oxygen species production. These findings suggest that CTS may play an anti-myocardial fibrosis role by inhibiting the phosphorylation of extracellular signal-regulated kinase 1/2 and the expression of COX-2, NOX-2, and NOX-4 induced by Ang II, improving pathological changes in the myocardium *in vivo* and reducing MF.

#### 4.5.2 *Salvia miltiorrhiza* Bunge extract

*S. miltiorrhiza* is a sterilized aqueous extract with various pharmacological properties, such as improved hemodynamics, anti-inflammatory effects, and antioxidant activity (Qiao et al., 2024) High-performance liquid chromatography can be used to identify three main metabolites: SAA (2.15 mg/mL), protocatechuic aldehyde (0.44 mg/mL), and Sal B (1.01 mg/mL). Danshen injection (3 g/kg/d or 6 g/kg/d) effectively prevents and treats oxidative stress injuries in zebrafish hearts and livers caused by iron overload. It reduces Hyp and MDA concentrations, lowers MMP-9 levels in mice, enhances SOD activity, and alleviates iron overload-related MF in a dose-dependent manner (Zhang, 2015).

#### 4.5.3 *Salvia miltiorrhiza* Bunge metabolite and its preparations

Metabolite Danshen Dripping Pills (CDSDP) (Yang et al., 2023a) can reduce ROS in the cardiac tissue of HF rats by inhibiting NRF2 expression and its target genes, leading to lower levels of NRF2, SOD2, and CAT in H9C2 and iPSC-derived cardiomyocytes and suppressing MF. CDSDP (660, 2,640 mpk) (Feng et al., 2021) enhances SOD1, p-AMPK, and NRF2 expression, reducing ROS and FFA levels. It also inhibits TGFβ1, αSMA, COL1A2, COL3A1, and MMP9 in myocardial tissue, decreasing MF.

Guanxin V (GXV) is a TCM formulation composed of *Codonopsis pilosula* (Franch.) Nannf. [Campanulaceae; Codonopsis Radix], *O. japonicus*, and other botanical drugs (Liang et al., 2020). Compared with the control, GXV significantly improved cardiac function in patients with coronary heart disease. A study (Liang et al., 2022) revealed that GXV (6 g/kg/d) decreased MDA and LDH levels in murine myocardial infarction models while increasing SOD, CAT, T-AOC, and GSH-Px levels, suggesting that GXV may reduce oxidative stress-related damage and inhibit MF.

Guanxintai (GXT) is a TCM approved by the CFDA in 1999 for managing coronary heart disease. Its botanical drugal formulations are composed of *P. ginseng*, *A. membranaceus*, *R. glutinosa*, *O. japonicus*, *S. chinensis*, *Boswellia sacra* Flück. [Burseraceae; Boswelliae Resina], *Commiphora myrrha* (T.Nees) Engl. [Burseraceae; Myrrha], *Angelica sinensis* (Oliv.) Diels [Apiaceae; Angelicae Sinensis Radix], C. anthriscoides, *Picrorhiza kurroa* Royle ex Benth. [Scrophulariaceae; Picrorhizae Rhizoma], *Achyranthes bidentata* Blume [Amaranthaceae; Achyranthis Bidentatae Radix], *S. miltiorrhiza*, *Acorus gramineus* Aiton [Acoraceae; Acori Graminei Rhizoma], and *L. japonicus*. Clinical studies indicate that GXT improves angina pectoris and arrhythmia and lowers blood lipid levels. A study (Yang et al., 2017) revealed that GXT (2 g/mL) reduces ROS levels and the expression of NOX and phosphorylated p38 MAPK proteins, leading to a decrease in cardiomyocyte injury and MF.

Danxiong Tongmai Granules (DXTMGs) are formulated from a combination of *P. lactiflora* Pall. [Paeoniaceae; Paeoniae Radix Alba], *C. anthriscoides*, *S. miltiorrhiza*, *Reynoutria multiflora* (Thunb.) Moldenke [Polygonaceae; Reynoutriae Multiflorae Caulis], *Corydalis yanhusuo* (Y. H. Chou & Chun C. Hsu) W. T. Wang ex Z. Y. Su & C. Y. Wu [Papaveraceae; Corydalis Rhizoma], *C. rotundus*, *C. tinctorius* and *Lycium chinense* Mill. [Solanaceae; Lycii Fructus]. Animal studies have indicated that 5 g of DXTMG significantly increases the serum levels of LDH, cTn-I, and MDA in heart failure model rats. It also increases SOD and GSH-PX levels while decreasing IL-6 and TNF-α levels and inhibiting MF (Ye et al., 2024). Table 5 summarizes *S. miltiorrhiza*’s antioxidative effects and underlying mechanisms in MF.

**TABLE 5:** Effective active metabolites and metabolite preparations of *Salvia miltiorrhiza* Bunge in inhibiting oxidative damage and improving MF.

Metabolite/metabolite	Experimental type	Model	Mechanism of action	Optimal dose	References
SAA	C57 mice	LAD	In myocardial tissue, SOD activity and collagen volume fraction decreased, while MDA levels increased	160 mg/kg/d	Liu and Wang (2021)
Tan IIA	Wistar rat	LAC	NADPH oxidase and SOD activity is reduced in myocardial tissue	15 mg/kg/d	Yuan et al. (2019)
Tan IIA	SD rats	LAC	HYPα-SMA, TGF-β1, HO-1, and MDA levels in myocardial tissue were downregulated	20 mg/kg/d	Cai et al. (2016)
Tan IIA	SD rats/CFs	LAD	TGF-β, α-SMA, MMP2, and MMP9 mRNA levels are decreased, while superoxide dismutase (Sod) activity is elevated in myocardial tissue	1.5 mg/kg/d	Chen et al. (2021)
STS	SD rats	Ang II	Myocardial tissue exhibits reduced CoL-I and CoL-III, along with lower levels of Keap1, cytoplasmic Nrf2 protein, and MDA.	20 mL/kg	Li et al. (2018a)
STS	CFs	Ang II	Inhibited ROS production	30 µM	Yang et al. (2009)
CTS	CFs	Ang II	Inhibiting the phosphorylation of extracellular signal-regulated kinase 1/2 and the expression of COX-2, NOX-2 and NOX-4 induced by Ang II	60 mg/kg/d	Ma et al. (2014)
Danshen injection	Zebrafish/Kunming mice	Water bath exposure/iron dextran	Zebrafish hearts exhibited increased levels of SOD and GSH, while MDA decreased. In mice, Hyp, MDA, and MMP-9 protein levels were reduced, and SOD activity increased	6 g/kg/d	Zhang (2015)
CDSDP	C57 mice/H9c2	WT, C57BL/6J), LDLRe/e) ApoEe/e	ROS levels in cardiac tissue and the expression of NRF2, SOD2, and CAT in H9c2 cells were reduced at both the protein and mRNA levels	—	Yang et al. (2023a)
CDSDP	C57 mice/H9c2	DOX/ISO	Suppression of TGFβ1, αSMA, COL1A2, COL3A1, and MMP9 in myocardial tissue decreases doxorubicin-induced ROS and MDA levels	2,640 mpk	Feng et al. (2021)
GXV	Syrian hamster/H9c2	LAD/H_2_O_2_	Myocardial tissue markers indicate decreased levels of MDA and LDH, while SOD, CAT, T-AOC, and GSH-Px are increased	6 g/kg/d	Liang et al. (2022)
GXT	Male wistar rat/H9c2	LAD	Inhibition of ROS, NOX and p38 MAPK protein expression	2 g/ml	Yang et al. (2017)
DXTMG	SD rats	ISO	The levels of LDH, cTn-I and MDA were decreased, and the levels of SOD and GSH-PX were increased	5 g/bag	Ye et al. (2024)

### 4.6 Anti-apoptotic action

Cardiomyocyte apoptosis promotes extracellular matrix remodeling and fibroblast activation in MF. Cardiomyocyte apoptosis is triggered by the interferon response of hosts or viral signals during myocarditis-related cardiac injury. This activates pathways such as the FAS/FASL, JAK-STAT, and mitochondria-dependent pathways, increasing the expression of proapoptotic molecules such as FasL, Fas, Bax, and cleaved Caspase-3. Caspase-3 activation is a critical apoptotic effector, mediating DNA fragmentation and cell death (Mazumder et al., 2008). Preclinical evidence (Yang et al., 2023b) demonstrates *S. miltiorrhiza* bioactives attenuate cardiomyocyte apoptosis via multiple pathway (Yang et al., 2008).

#### 4.6.1 Effectively active metabolites of *Salvia miltiorrhiza* Bunge

Chen Y. F. et al. (2017) reported that Tan IIA downregulates proteins such as MMP-9, MMP-2, TGF-β1, p-Smad2/3, SP-1, and CTGF in cardiomyocytes. It also upregulates TIMP-1 and TIMP-2 while lowering Caspase-3 and Caspase-9 levels, leading to a decrease in cardiomyocyte apoptosis and MF.

Sal A is a hydrophilic metabolite derived from *S. miltiorrhiza* that has several pharmacological properties, including antioxidant and anti-fibrotic effects. Anwaier et al. (2022) reported that administering various doses of Sal A (10 mg/kg/d, 20 mg/kg/d, and 40 mg/kg/d) via intraperitoneal injection enhances cardiac function in rats with DOX-induced MF. These findings indicated a reduction in the serum levels of tumor necrosis factor-α (TNF-α), homocysteine (Hcy), and endothelin (ET), as well as a decrease in galectin-3 and TGF-β/Smad protein expression in myocardial tissue, significantly reducing myocardial cell apoptosis, thereby inhibiting MF, and that high-dose Sal A treatment is optimal.

#### 4.6.2 *Salvia miltiorrhiza* Bunge extract

DHI intervention for 4 weeks significantly reduces myocardial interstitial collagen density in a rat model of heart failure. It suppresses TGF-β1, MMP-2, MMP-9, and Caspase-3 while enhancing Bcl-2 expression, improving cardiac function, and inhibiting MF (Chen et al., 2016).

#### 4.6.3 S*alvia miltiorrhiza* Bunge metabolite and its preparations

Lichan Tao and colleagues reported that (Tao et al., 2015) QLQXC (0.5 g/kg/d) significantly reduced CoL-I, CoL-III, and α-SMA levels in a rat model of heart failure. QLQXC treatment also decreased the expression of TGF-β1, MMP-2, and MMP-9 and the Bax/Bcl-2 ratio, indicating that it can protect the myocardial cell structure and reduce MF.

Lv et al. (2022) reported that at doses of 135 mg/kg/d, 270 mg/kg/d, and 540 mg/kg/d, QSYQDP significantly reduced CoL-I and CoL-III in the myocardial tissue of rats with autoimmune cardiomyopathy. This occurred due to the downregulation of Bcl-2, upregulation of Bax and caspase-3, and inhibition of myocardial cell apoptosis, resulting in a decrease in MF, particularly in the high-dose group. Another study (Anwaier et al., 2022) reported that QSYQDP (0.8 g/kg/d) effectively decreased serum NT-ProBNP, LDH, and cardiac MDA levels in rats with HF from aortic coarctation. It also decreased the ratios of caspase-3, caspase-9, Bax, and Bcl-2, inhibited cardiomyocyte apoptosis, and enhanced myocardial function. Wang L. et al. (2019) reported that administering 5 mg/kg/d QSYQDP for 4 weeks significantly reduced Bax, caspase-3, Bcl-2, and α-SMA protein levels in the myocardial tissue of rats with doxorubicin-induced HF, leading to a decrease in myocardial apoptosis and the inhibition of fibrosis.

Guanxinning tablet (GXN), derived from *S. miltiorrhiza* and *C. anthriscoides* at a 1:1 ratio, show significant pharmacological efficacy in preventing and managing cardiovascular diseases (Hu et al., 2017). These metabolites effectively suppress sympathetic nerve activity, enhance left ventricular function, improve hemorheological abnormalities, increase plasma NO levels in rat models, and provide therapeutic benefits for myocardial ischemia. Zhang Y. et al. (2023) reported that GXN at doses of 600 mg/kg/d and 1,200 mg/kg/d effectively inhibited cardiomyocyte apoptosis, reduced Bax and Bcl-2 mRNA and protein levels, and suppressed myocardial remodeling and fibrosis in rats with heart failure induced by aortic coarctation.

Liang et al. (2022) demonstrated that GXV (0.25 g/L, 0.50 g/L, 0.75 g/L, and 1 g/L) effectively inhibited cardiomyocyte apoptosis and MF in a dose-dependent manner.

The newly optimized formulation of Shengmaisan (NO-SMS) is derived from the traditional Shengmaisan recipe. This formulation is processed into granules through a series of methods, including boiling, extraction, and vacuum techniques, following a specific ratio of 10:10:10:10:6:10:6:10:6:10. The metabolites used in this formulation are composed of *A. membranaceus*, *C. pilosula*, *Eleutherococcus senticosus* (Rupr. & Maxim.) Maxim. [Araliaceae; Acanthopanacis Senticosi Radix et Rhizoma], *Tinospora crispa* (L.) Hook. f. & Thomson [Menispermaceae; Tinosporae Caulis], *D. sophia*, *P. cocos*, *C. × aurantium* f. aurantium, and *S. miltiorrhiza*. The active metabolites identified in this optimized formulation include isorhamnetin, quercetin, kaempferol, and tanIIA (Hou et al., 2023) (0.81 g/kg/d), which significantly reduce myocardial cell apoptosis in rats with HF. This treatment downregulated proteins such as Caspase-3, Caspase-9, Caspase-12, Cyt-c, and Bax while increasing the Bcl-2/Bax ratio, thus inhibiting apoptosis and mitigating MF. Table 6 summarizes *S. miltiorrhiza*’s anti-apoptotic effects and underlying mechanisms in MF.

**TABLE 6:** Effective active metabolites and metabolite preparations of *Salvia miltiorrhiza* Bunge in anti-apoptotic action and improving MF.

Metabolite/metabolite	Experimental type	Model	Mechanism of action	Optimal dose	References
Tan IIA	H9c2	Ang II	Downregulation of MMP-9, MMP-2, TGF-β1, p-Smad2/3, SP-1 and caspase-3, caspase-9 protein in cardiomyocytes	—	Chen et al. (2017b)
Sal A	SD rats	DOX	Reduce the expression of Galectin-3 and TGF-β/Smads proteins in myocardial tissue	40 mg/kg/d	Ai et al. (2022)
DHI	SD rats	LAD	The expression of TGF-β1, MMP-2, MMP-9 and Caspase-3 was downregulated, and the expression of Bcl-2 was upregulated	—	Chen et al. (2016)
QLQXC	C57 mice	LAD	The expression of a-SMA, TGFb1, MMP-2, MMP-9 and the ratio of Bax/Bcl-2 were downregulated, and Smad7 was upregulated	0.5 g/kg/d	Tao et al. (2015)
QSYQDP	Lewis male rats	AIM	The content of Bax and caspase-3 protein in myocardial tissue was decreased, and the expression of Bcl-2 was increased	540 mg/kg/d	Lv et al. (2022)
QSYQDP	SD rats/H9c2/CFs	COA/Ang II	The expression of caspase-3, caspase-9 and Bax/Bcl-2 were decreased	0.8 g/kg/d	Anwaier et al. (2022)
QSYQDP	C57 mice	DOX	Reduce the expression of Bax, caspase-3, Bcl-2 and a-SMA protein in myocardial tissue and reduce myocardial apoptosis	5 mg/kg/d	Wang et al. (2019a)
GXN	SD rats/CFs	COA	BAX mRNA was downregulated and BCL2 mRNA and protein expression was upregulated	1,200 mg/kg/d	Zhang et al. (2023b)
GXV	Syrian hamster/H9c2	LAD/H_2_O_2_	Hoechst 33342 staining showed that it inhibited cardiomyocyte apoptosis	1 g/L	Liang et al. (2022)
NO-SMS	SD rats/H9c2	LAD	The expression of Caspase-3, Caspase-9, Caspase-12, Cyt-c and Bax was downregulated, and the ratio of Bcl-2/Bax was increased	0.81 g/kg/d	Hou et al. (2023)

### 4.7 Inhibition of the proliferation of CFs

Following cardiac injury, fibroblasts activate and differentiate into myofibroblasts, which secrete excessive ECM components (e.g., collagen) to repair the myocardium. Excessive activation can cause the accumulation and remodeling of the ECM, impairing cardiac function and potentially leading to heart failure (Frangogiannis, 2021). The abnormal proliferation of CFs is linked to several cardiovascular disorders, such as hypertension and myocardial infarction. Regulating this proliferation is essential for managing MF. *S. miltiorrhiza* and its bioactive metabolites can reduce MF by inhibiting fibroblast proliferation and collagen fiber formation (Yang et al., 2022).

#### 4.7.1 Effective active metabolites of *Salvia miltiorrhiza* Bunge

Different concentrations of Tan IIA (0.01 mmol/L, 0.1 mmol/L, and 1 mmol/L) reduce the proliferation rate, Hyp levels, and mRNA expression of CoL-I, CoL-III, and TIMP-2 in Ang II-induced CFs (Wang S. et al., 2019). Fu and Sun (2016) reported that Tan IIA (10–6 mol/L) could reduce the proliferation rate of Ang II-induced CFs and the content of CoL-I, increase the percentage of CFs in the G0/G1 phase, and decrease the percentage of cells in the S phase. Additionally, Tan IIA inhibited the protein expression of PKC and cyclin D1, indicating that Tan IIA significantly suppressed CF proliferation and collagen secretion by modulating the PKC-cyclin D1 signaling pathway. Chan et al. (2011) reported that Tan IIA, at concentrations ranging from 3 to 10 μM, effectively inhibited the proliferation of CFs and reduced the ROS levels induced by Ang II.

Han et al. (2022) reported that different concentrations of Sal A (25, 50, and 100 mg/L) inhibited the proliferation of CFs induced by high glucose and reduced the secretion of Col-I and Col-III in CFs. Sal A significantly inhibited the expression of TGF-β1 and β-catenin but upregulated the expression of p-GSK-3β at a concentration of 100 mg/L. These results suggest that Sal A inhibits CF proliferation by modulating the Wnt signaling pathway.

Wang et al. (2020) reported that Sal B (12.5 μmol L^–1^, 25 μmol L^–1^, and 50 μmol L^–1^) effectively inhibited the proliferation of CFs stimulated with high glucose levels and reduced α-SMA, β-catenin, and p-GSK 3β protein expression. These findings suggest that Sal B may impede CF proliferation and transdifferentiation by modulating the Wnt/β-catenin signaling pathway, enhancing MF. Huang et al. (2017) reported that Sal B at concentrations of 2.5 × 10^−5^, 5 × 10^−5^, and 5 × 10^−4^ mol L^–1^ effectively reduced Ang II-induced proliferation of CFs. This treatment decreased Hyp levels and downregulated collagen type I and alpha-smooth muscle actin expression, alleviating MF. Luo et al. (2014) reported that Sal B at concentrations of 10 μmol/L, 30 μmol/L, and 100 μmol/L effectively inhibited TGF-β1-induced CF proliferation, with the highest concentration having the most significant effect on enhancing MF. Wang et al. (2011) reported that Sal B (0.01 µM, 0.1 µM, 1 μM, and 10 µM) significantly inhibited the MMP9-induced proliferation of CFs and their transformation into myofibroblasts, providing protective effects for the heart. Lu et al. (2024) conducted *in vivo* and *in vitro* experiments and revealed that Sal B (20 mg/kg/d and 40 mg/kg/d) effectively inhibited the aberrant expression of TRPC6. Downregulation of Smad3 inhibited the proliferation of fibroblasts, reduced the activation of the TGF-β/Smad3 signaling pathway, and alleviated diabetic MF. Sun (2017) Huangqi Baoxin Decoction (HQBXD) (15 g/kg) and its active metabolite Sal B (5 mol/L, 6 mol/L, and 7 mol/L) effectively mitigate Ang II-induced proliferation and collagen synthesis in CFs. This effect is achieved through the inhibition of the TGF-β/Smad signaling pathway, which decreases the expression levels of Col I and Col III mRNAs, as well as the proteins galectin-3, TGF-β, and Smad3.

#### 4.7.2 *Salvia miltiorrhiza* Bunge metabolite and its preparations

Sun et al. (2023) reported that different doses of QLQXC (0.1 mg/mL and 0.5 mg/mL) could downregulate the expression of CoL-I, CoL-III, PAI-1, TGFβ1, and p-Smad proteins to different degrees, indicating that QLQXC could inhibit the activation of CFs induced by adriamycin by inhibiting the PAI-1/TGFβ1/Smad3 pathway.

QSG comprises six botanical drugs (*A. membranaceus*, *S. miltiorrhiza*, *A. carmichaelii*, *S. ningpoensis*, *G. glabra*, and *L. japonica* and comes from two well-known TCM formulae, namely, “Zhen Wu Decoction” and “Simiao Yongan Decoction.” QSG (Tan et al., 2022) (1 mg/mL) inhibited the proliferation of CFs and reduced the content and mRNA expression of Col-I and Col-III.

The formulation of Yixintai Granules (YXTG) is composed of *A. membranaceus*, *C. tinctorius*, *S. miltiorrhiza*, *A. orientale*, *P. umbellatus*, and *D. sophia* at proportions of 3:1:1:2:1:1. The preparation process involves extraction, filtration, and concentration to create granules, which are commonly used in clinical settings for treating CHF. Yang et al. (2021) reported that plasma containing YXTG significantly inhibits the proliferation of CFs induced by Ang II, causing cell cycle arrest in the G0/G1 phase and downregulating the mRNA levels of PCNA, α-SMA, Col-I, and Col-III. YXTG was also shown to impede the proliferation, differentiation, and fibrotic activity of CFs.

Yixintai (YXT) is formulated from a combination of *A. membranaceus*, *S. miltiorrhiza*, *C. tinctorius*, *A. orientale*, and *Waltheria indica* L. [Sterculiaceae; Waltheriae Indicae Herba] at proportions of 3:1.5:1:1:1. YXT is prepared by producing a dry powder from its alcohol extract through filtration, vacuum concentration, and drying. Studies have indicated that YXT can improve ventricular remodeling and cardiac function in CHF models in rats and rabbits (Tang and Guo, 2020a; Tang and Guo, 2020b; Tang et al., 2015). Tang et al. (2021) reported that YXT alcohol extracts (50, 100, and 200 mg/L) significantly reduced the viability of CFs from neonatal rabbits stimulated with Ang II, inhibited CF proliferation, and suppressed myofibroblast differentiation.

The Huoxue Qianyang Qutan Recipe (HQQR) was formulated via a combination of *S. miltiorrhiza*, *Concha Ostreae* [Ostreidae; Concha Ostreae], *C. anthriscoides*, *Uncaria rhynchophylla* (Miq.) Miq. [Rubiaceae; Uncariae Ramulus cum Uncis], *Taxillus chinensis* (DC.) Danser [Loranthaceae; Taxilli Ramulus], *C. monogyna*, and *Curcuma zedoaria* (Christm.) Roscoe [Zingiberaceae; Curcumae Zedoariae Rhizoma] at a drug ratio of 9:20:9:15:15:9:30. *In vitro* studies (Lu et al., 2024) indicated that HQQR doses of 1 mg/mL and 1.25 mg/mL effectively reduced Ang II-stimulated CF proliferation, decreased Hyp levels, and increased MF, with higher doses showing greater efficacy.

Yiqi Huoxue Decoction (YQHXD) Cui et al. (2021), derived from the Buyang Huanwu Decoction, consists of *P. ginseng*, *A. membranaceus*, *P. lactiflora*, *C. anthriscoides*, *J. regia*, *C. tinctorius*, *P. notoginseng*, *S. miltiorrhiza*, *Curcuma aromatica* Salisb. [Zingiberaceae; Curcumae Aromatica Rhizoma], *C. reticulata*, *Dolomiaea costus* (Falc.) Kasana & A.K.Pandey [Asteraceae; Dolomiaeae Costus], *P. odoratum* (Mill.) Druce [Asparagaceae; Polygonati Odorati Rhizoma], and *L. chinense* Mill. [Solanaceae; Lycii Fructus] at ratios of 3:20:5:5:5:5:2:2:2:2:2:2 and is prepared as a freeze-dried powder. Various concentrations of YQHXD (40 μg/mL, 80 μg/mL, and 160 μg/mL) effectively reduced Col-I and Col-III protein levels in CFs, inhibited their proliferation, and protected cardiomyocytes.

Fuzheng Huayu Decoction (FZHYD) is formulated with a combination of *S. miltiorrhiza*, *C. mycelium*, *G. pentaphyllum*, *P. massoniana*, and *S. chinensis* and adheres to a specific ratio of 8:4:2:6:2:2. Zhu et al. (2019a) demonstrated that FZHYD at concentrations of 25 μg/mL, 50 μg/mL, and 100 μg/mL inhibited Ang II-induced proliferation of CFs in a dose-dependent manner.

Jiashenfang (JSF) is an effective treatment for CHF. This herbal formula consists of the following botanicals: *Periploca sepium* Bunge [Apocynaceae; Periplocae Cortex], *P. notoginseng*, *A. membranaceus*, *SM*, *L. japonicus*, *D. sophia*, *P. cocos*, and *A. orientale*. Following concentration screening, Cui et al. (2016) reported that the viability of CFs was not adversely affected by the application of 0.25 mg/mL JSF extract. Subsequent experimental investigations using the same concentration of JSF extract revealed its ability to decrease the fluorescence intensity of α-SMA and reduce the expression of Hyp in CFs stimulated with TGF-β1.

The Bushen Huoxue Decoction (BSHXD) is composed of the following botanicals: *C. officinalis*, *Cistanche deserticola* Ma [Orobanchaceae; Cistanches Herba], *P. lactiflora*, *C. anthriscoides* and *S. miltiorrhiza*. Specific dosages are not provided in this study. Ma X. et al. (2012) studied SD rats given 800 mg/d BSHXD for 4 days to obtain enriched serum. The results showed that 10% and 20% concentrations of this serum significantly inhibited CF proliferation and reduced Col-I and Col-III levels. SM inhibited the proliferation of CFs as shown in Table 7.

**TABLE 7:** Effective active metabolites and metabolite preparations of *Salvia miltiorrhiza* Bunge in inhibition of the proliferation of CFs and Improving MF.

Metabolite/metabolite		Experimental type		Model		Mechanism of action		Optimal dose		References	
Tan IIA		CFs		Ang II		The rate of cell proliferation, along with the expression levels of Hyp, Col I, Col III, and TIMP-2 mRNA, exhibited a downregulation		1 mmol/L		Wang et al. (2019b)	
Tan IIA		CFs		Ang II		The rate of proliferation of CFs was suppressed, and there was a reduction in the expression levels of Col I		10^−6 ^mol/L		Fu and Sun (2016)	
Tan IIA		CFs		Ang II		Suppress the proliferation rate of CFsand decrease the levels of ROS.		10 μM		Chan et al. (2011)	
Sal A		CFs		High glucose induction		Inhibited the expression of TGF-β1 and β-catenin, while upregulating the expression of p-GSK-3β, inhibited the proliferation of CFs		100 mg/L		Han et al. (2022)	
Sal B		CFs		High glucose induction		The proliferation of CFs was inhibited, and the protein levels of α-SMA, β-catenin and p-GSK 3β were decreased		50 μmol L^−1^		Wang et al. (2020)	
Sal B		CFs		Ang II		The abnormal proliferation and Hyp content of CFs were decreased, and the expression of Col I and α-SMA protein was downregulated		10^−4^ mol L^−1^		Huang et al. (2017)	
Sal B		CFs		TGF-β1		Inhibition of CFs proliferation rate		100 μmol/L		Luo et al. (2014)	
Sal B		CFs		MMP-9		Inhibit the transformation of cardiac fibroblasts into myofibroblasts phenotype		10 μM		Wang et al. (2011)	
Sal B		C57BL/6J mice/CFs		STZ + 30% high fat diet feeding		Inhibit the proliferation of fibroblasts, reduces the activation of the TGF-β/Smad3 signaling pathway		40 mg/kg/d		Lu (2024)	
Sal B		CFs		Ang II		Downregulation of Galectin-3, TGF-β, and Smad3 protein expression in CFs		7 mol/L		Sun (2017)	
QLQXC		CFs		DOX		Downregulation of Col I, Col III, PAI-1, TGFβ1, p-Smad protein expression, inhibition of CFs activation		0.5 mg mL^−1^		Sun et al. (2023)	
QSG		CFs		Ang II		The proliferation of CFs was inhibited, and the content and mRNA expression of Col I and Col III were decreased		1 mg/mL		Tan et al. (2022)	
YXTG		CFs		Ang II		The proliferation of CFs was inhibited, and the expression of PCNA, α-SMA, Collagen I and Collagen III mRNA was downregulated		2.6 g kg^−1^		Yang et al. (2021)	
Yixintai alcohol extract		Bunny CFs		Ang II		Reduce CFs cell viability and inhibit CFs proliferation		200 mg/L		Tang et al. (2021)	
HQQR		CFs		Ang II		Inhibition of CFs proliferation and Hyp content		1.25 mg/ml		Lu et al. (2024)	
YQHXD		CFs		high glucose induction		The expression of Col I and Col III protein in CFs was decreased, and the proliferation of CFs was inhibited		160 μg/ml		Cui (2021)	
FZHYD		CFs		Ang II		Inhibition of Ang II-induced CFs proliferation		100 μg/ml		Zhu et al. (2019a)	
JSF		CFs		Ang II		The proliferation rate of CFs was inhibited, and the content of α-SMA and Hyp was decreased		0.25 mg/mL		Cui et al. (2016)	
BSHXD		CFs		Ang II		Inhibit the proliferation rate of CFs and reduce the expression of Col I and Col IIII protein		20% medicated serum		Ma et al. (2012b)	

## 5 Clinical utilization of *Salvia miltiorrhiza* Bunge in the prevention and management of MF

Many researchers have focused on the basic use of *S. miltiorrhiza* in the treatment of MF, and several researchers have focused on its clinical application. To further verify its anti-MF effect and safety, researchers have conducted many randomized controlled trials (RCTs). Several experiments have shown that *S. miltiorrhiza* and its metabolite preparations have better clinical efficacy in combination with biomedicine medicine and that the two play a synergistic role.

### 5.1 Active metabolites of *Salvia miltiorrhiza* Bunge

A clinical study retrospectively analyzed the medical records of 80 patients after PCI (Deng, 2023). The control group was treated with ivabradine hydrochloride tablets, while the observation group was subjected to STS injection (40 mg/d) on the basis of the control group. After 2 weeks of continuous treatment, the total effective rate of treatment in the observation group was 92.50%, and that in the control group was only 75.00%. The levels of serum CTGF, sST2, TGF-β1, and Gal-3 decreased, and the effect was greater than that of simple ivabradine treatment. These findings indicate that the combination of STS with ivabradine for the treatment of patients after PCI can improve the total effective rate of treatment, improve cardiac function, reduce MF, and improve the effect of simple ivabradine treatment.

### 5.2 *Salvia miltiorrhiza* Bunge extract

Chen Z. et al. (2019) collected 176 patients with AMI and randomly divided them into a control group and an observation group, with 88 patients in each group. The control group was given alteplase for injection on the basis of routine treatment, whereas the observation group was given DHI on the basis of the control group. After 2 weeks of continuous treatment, the levels of Gal-3, TGF-β1, CTGF, NF-κB, cystatin C, MMP-9, and FGF-23 in the two groups significantly decreased, and those in the treatment group were significantly greater than those in the control group. The total effective rate was 74% in the control group and 83% in the observation group. After 6 months of follow-up, there were 2 cases of MACE in the observation group and 6 cases in the control group. DHI combined with alteplase was helpful in inhibiting MF and ventricular remodeling, and the effect was better than that of alteplase alone.

### 5.3 *Salvia miltiorrhiza* Bunge metabolite and its preparations

Xu et al. (2019) used rosuvastatin calcium tablets combined with metabolite Danshen dripping pills (FFDSDP) (10 pills/time, 3 times/d) for 24 weeks to treat patients after PCI. The serum levels of MMP-9, TGF-β1, and CTGF in the observation group were lower than those in the control group, and the total effective rate was 39% in the observation group and 33% in the control group. During the treatment period, there were no obvious adverse reactions in the two groups, indicating that the application of rosuvastatin combined with FFDSDP can better inhibit MF and improve the clinical symptoms of patients.

Li and Sun (2023) reported that a QLQXC (1.2 g/time, 3 times/d) combined with levosimendan for 3 months could reduce the levels of NT-pro BNP, cTnI, cTnTLN, HA and PCIII in elderly patients with CHF and can also reduce the expression of serum CRP, IL-6 and TNF-α. The clinical efficacy of the observation group (95%) was better than that of the control group (77.5%), indicating that QLQXC combined with levosimendan can improve the clinical effect, reduce myocardial injury in patients, promote the downregulation of inflammatory factors, improve MF, and alleviate clinical symptoms.

CHF patients (Xu Q. et al., 2024) were treated with Shengmai Qiangxin granules (SMQXG) (1 bag/time, 3 times/day) on the basis of conventional biomedicine medicine for 4 weeks. After 4 weeks of treatment, the levels of serum TGF-β1, MMP-2, and PIIINP decreased, and the expression of TLR4, MyD88, and NF-κB mRNA and protein decreased. The total effective rate of the conventional biomedicine medicine treatment group was 26%, and the total effective rate of the control group was 36%. There was no significant difference in the incidence of adverse reactions between the two groups, indicating that SMQXG inhibited MF by regulating TLR4/MyD88/NF-κB signaling and alleviated the clinical symptoms of patients. In addition, the safety is good.

Shenfu Yixin Decoction (SFYXD) is formulated with the following metabolites: *S. miltiorrhiza*, *C. cassia*, *Zingiber officinale* Roscoe [Zingiberaceae; Zingiberis Rhizoma], *A. orientale*, *A. membranaceus*, *D. sophia*, *C. rotundus*, *P. ginseng*, *A. macrocephala*, *P. cocos*, *G. glabra*, *Plantago asiatica* L. [Plantaginaceae; Plantaginis Semen], and *Areca catechu* L. [Arecaceae; Arecae Semen], at ratios of 20:10:6:10:20:10:15:8:20:15:9:10:10. Clinical studies (Wang Y. et al., 2024) have shown that the basic treatment of biomedicine medicine combined with SFYXD for 2 months to treat HFrEF results in lower serum levels of sCD146, NT-proBNP, cTnI, Gal-3, Ang II, CgA and sST2 than those in the basic treatment group of biomedicine medicine, and PCO2, DO2, VO2 and LVEF are higher than those in the basic treatment group of biomedicine medicine. The total effective rate of SFYXD combined with biomedicine medicine was 98.41%, whereas that of the biomedicine medicine treatment group was only 84.13%, indicating that SFYXD can promote the repair of myocardial injury, regulate the oxygen dynamics index and reduce MF. The effect was better than that of pure biomedicine medicine.

The Yiqi Huoxue prescription (YQHXP) consists of *A. membranaceus*, *S. miltiorrhiza*, *Rhodiola rosea* L. [Crassulaceae; Rhodiolae Radix et Rhizoma], *C. pilosula*, *C. cassia*, *P. lactiflora*, *O. japonicus*, *S. chinensis* and *Vitex negundo* L. [Verbenaceae; Viticis Negundi Herba]. The formula ratio is 20:20:15:10:3:10:10:10:10:10. It is commonly used for the clinical treatment of HF. Zhang C. et al. (2023) selected 41 patients with acute ST-segment elevation myocardial infarction and divided them into a control group (n = 20) and an observation group (n = 21). The control group was given aspirin + ticagrelor + atorvastatin calcium tablets. Angiotensin-converting enzyme inhibitors (ACEIs), angiotensin receptor enkephalinase inhibitors (ARNI), β-receptor blockers (β-RBs), and aldosterone receptor antagonists were used to reduce blood pressure and heart rate. For the standard treatment of myocardial infarction, the observation group was treated with YQHXP on this basis, and the course of treatment was 12 weeks. Through clinical research, YQHXP combined with conventional drugs after 12 weeks of treatment was shown to reduce the levels of Lp-PLA2, hs-CRP, IL-6, NT-proBNP, ECV, and T1 in patients with heart failure, and the major cardiovascular adverse events of the two groups were not statistically significant, indicating that YQHXP can improve the clinical symptoms of patients with acute ST-segment elevation myocardial infarction after PCI and inhibit myocardial fibrosis. The mechanism may be related to the reduction in the inflammatory response.

The Shugan Yixin prescription (SGYXP) is composed of the following botanicals: *Bupleurum chinense* DC. [Apiaceae; Bupleuri Radix], *S. miltiorrhiza*, *P. ginseng*, *Pinellia ternata* (Thunb.) Makino [Araceae; Pinelliae Rhizoma], *Moringa oleifera* Lam. [Moringaceae; Moringae Oleiferae Folium], *Scutellaria baicalensis* Georgi [Lamiaceae; Scutellariae Radix], *Prunus armeniaca* L. [Rosaceae; Armeniacae Semen], *C. chinensis* Franch. [Ranunculaceae; Coptidis Rhizoma], *P. cocos*, *C. yanhusuo* (Y. H. Chou & Chun C. Hsu) W. T. Wang ex Z. Y. Su & C. Y. Wu [Papaveraceae; Corydalis Rhizoma], and *G. glabra*, at ratios of 15:20:10:10:12:12:10:6:12:10:6. Gan et al. selected 82 patients with coronary heart disease and divided them into a control group and an observation group, with 41 patients in each group. The control group was given isosorbide dinitrate tablets + enalapril maleate tablets + digoxin tablets + metoprolol tartrate tablets + aspirin enteric-coated tablets. The observation group was treated with SGYXP on the basis of the control group. The treatment period was 4 weeks. After 4 weeks, the corresponding indicators were detected (Gan et al., 2020). After 4 weeks of continuous intervention, SGYXP significantly reduced the serum BNP, CRP, HA, and PCIII levels in patients with coronary heart disease. The effective rate of the observation group was 35%, and the effective rate of the control group was 19%. These findings indicate that SGYXP has a significant effect on the treatment of coronary heart disease and can improve MF and promote the recovery of cardiac function.

Shenyuan Yiqi Huoxue Decoction (SYYXHXD) is composed of the following components: *A. membranaceus*, *S. miltiorrhiza*, *C. pilosula*, *S. ningpoensis*, *C. yanhusuo*, *Aloe vera* (L.) Burm. f. [Asphodelaceae; Aloes], *Eupolyphaga seu Steleophaga (ground beetle)*, *Hirudo (leech)* at a ratio of 30:30:15:15:10:10:6:3. In randomized controlled trials, Chu et al. (2020) 84 patients with coronary heart disease and heart failure were randomly divided into a treatment group and a control group, with 42 cases in each group. The control group was given routine treatment, such as antiplatelet aggregation, statins, β-blockers, nitrates, angiotensin converting enzyme inhibitors/angiotensin II receptor antagonists, and diuretics. The treatment group was given SYYXHXD on the basis of the control group. The results revealed that the levels of plasma thrombin, TGF-β1, NT-proBNP, serum Col-I, and Col-III were significantly decreased in the treatment group. Compared with those before treatment, the blood, urine, and stool parameters and liver and kidney function of the two groups were not significantly aggravated. These findings indicate that SYYXHXD combined with conventional biomedicine medicine can reduce the MF level in coronary heart disease patients and that the effect is better than that of biomedicine medicine alone.

Zhenwu Baoxin Decoction (ZWBXD) contains processed *C. rotundus*, *P. cocos*, *W. indica*, *P. lactiflora*, *A. macrocephala*, *Z. officinale*, *C. cassia*, *S. miltiorrhiza*, and *G. glabra* at a ratio of 9:9:9:9:15:5:10:15:10. Yan (2019) randomly divided 83 patients with CHF into a control group and an observation group. Forty-one patients in the control group were treated with telmisartan tablets + spironolactone tablets, and 42 patients in the observation group were treated with ZWBXD on the basis of biomedicine medicine. After 4 weeks of continuous treatment, the levels of serum PCI, PCIII, HA and LN in CHF patients decreased, the LVEF, SV, and 6 MWT increased, and those in the observation group were better than those in the control group, indicating that ZWBXD combined with biomedicine medicine can effectively improve the cardiac function of CHF patients and prevent MF and that the curative effect is better than that of biomedicine medicine alone.

Jiawei Wendan Decoction (JWWDD) contains: *Trichosanthes kirilowii* Maxim. [Cucurbitaceae; Trichosanthis Fructus], *S. miltiorrhiza*, *C. pilosula*, *Phyllostachys nigra* var. henonis (Mitford) Rendle [Poaceae; Phyllostachydis Henonensis Caulis], *Smilax glabra* Roxb. [Liliaceae; Smilacis Glabrae Rhizoma], *P. ternata*, *C. reticulata*, and *C. anthriscoides* at ratios of 20:20:15:15:10:10:10:10. Xie et al. divided patients after PCI into an observation group and a control group. The observation group was treated with low-molecular-weight heparin calcium injection + aspirin enteric-coated tablets + clopidogrel bisulfate tablets + atorvastatin calcium tablets. The treatment group was combined with JWWDD on the basis of the observation group. After 6 months of continuous treatment, the levels of PCI, HA, PCIII, and LN in patients after PCI significantly decreased, the SAQ score significantly increased, and the LVEF, CO, and SV significantly increased compared with those before treatment. The clinical efficacy of the control group (70.15%) was better than that of the observation group (88.24%). This finding shows that Jiawei Wendan decoction can reduce the degree of MF in patients after PCI and enhance their cardiac function, and the effect is significant (Xie et al., 2018).

The Yiqi Huayu Decoction (YQHYD) combines Baoyuan and Xuefu Zhuyu decoctions, consisting of *A. membranaceus*, *C. chinensis*, *A. sinensis*, *D. sophia*, *C. anthriscoides*, *Panax quinquefolius* L. [Araliaceae; Panax Quinquefolii Radix], *A. vera*, *P. lactiflora*, *J. regia*, *S. miltiorrhiza*, *leech*, *L. japonicus*, and *C. cassia*, at ratios of 30:6:10:10:15:15:15:15:15:15:12:20:5:15:30:5. In one study (Cui et al., 2017), 154 patients with CHF were randomly divided into a control group and an observation group. The control group was treated with telmisartan tablets, metoprolol tartrate tablets and spironolactone. Compared with the control group, the observation group was treated with YQHYD (1 dose per day) for 3 months. The results revealed that the serum levels of GF-β1, CTGF, MMP-2, HA, PCIII, LN and PCI in CHF patients were decreased, and the clinical efficacy of the control group (50%) was better than that of the observation group (63%), indicating that CHF combined with conventional biomedicine medicine plus YQHYD can improve clinical symptoms and improve cardiac function; the mechanism may be related to the reduction in MF.

Yiqi Yangyin Huoxue Decoction (YQYYHXD) is composed of the following components: *A. membranaceus*, *C. pilosula*, *O. japonicus*, *S. chinensis*, *C. cassia*, *R. glutinosa*, *S. miltiorrhiza*, *C. anthriscoides*, *L. japonicus*, and *G. glabra* at ratios of 30:15:20:10:10:30:25:15:15:12. Lv’s team (Lu G. et al., 2022) randomly divided 82 CHF patients into a control group and a study group, with 41 patients in each group. The control group was treated with telmisartan tablets, metoprolol tartrate tablets, and spironolactone. On this basis, the study group was treated with YQYYHXD. The course of treatment in both groups was 3 months. The results revealed that the levels of LVEF, SV, E/A, and the 6 MWT in the two groups were significantly increased, the levels of plasma NT-proBNP were significantly decreased, and the levels of serum IL-1β, hs-CRP, TNF-α, MMP-9, TGF-β1, PCIII, sST2 and galectin-3 were decreased. The clinical efficacy of the control group (70.7%) was better than that of the observation group (90.2%), indicating that YQYYHXD could relieve the clinical symptoms of patients and inhibit MF. The mechanism may be related to the inhibition of the inflammatory response.

The Baoxin mixture (BXHJ) is composed of the following components: *A. membranaceus*, *C. pilosula*, *Testudinis Plastrum*, *S. miltiorrhiza*, *Forsythia suspensa* (Thunb.) Vahl [Oleaceae; Forsythiae Fructus], *C. cassia*, *S. glabra*, *Triticum aestivum* L. [Poaceae; Triticus Aestivus], and *S. chinensis*, at ratios of 40:20:15:15:15:15:6:15:15:6.

Bai’s team (Bai et al., 2021) randomly divided 100 patients with heart failure into a control group and a treatment group, with 50 patients in each group. The control group was treated with diuretics, antiplatelet aggregation, lipid-lowering and plaque-stabilizing agents, aldosterone receptor antagonists, ARNI, β-receptor blockers and other conventional biomedicine medicines for heart failure. The treatment group was treated with the BXHJ on this basis. Both groups were treated for 12 days. The results revealed that the levels of plasma IL-1β, TNF-ɑ, IL-6, CRP, sST2, and Gal-3 in the two groups were decreased, and the combined treatment effect was better than that of conventional biomedicine medicine.

Yiqi Yangyin Huoxue Buxin Decoction (YQYYHXBXD) is formulated at a ratio of 30:10:15:9:20:9:20:9:20:6:6:12:6:12 and comprises *A. membranaceus*, *P. ginseng*, *O. japonicus*, *S. chinensis*, *S. miltiorrhiza*, *P. lactiflora*, *R. glutinosa*, *Colla Corii Asini*, *C. cassia*, *Cannabis sativa* L. [Cannabaceae; Cannabis Fructus], *P. cocos*, and *G. glabra*. In one study, 88 elderly patients with CHF were divided into a control group and an observation group by the random number table method, with 44 patients in each group. The control group was treated with conventional biomedicine medicine, and the observation group was treated with YQYYHXBXD on the basis of the control group for 12 weeks (Zhang and Wei, 2021). YQYYHXBXD combined with biomedicine medicine can reduce the levels of serum sST2, galectin-3, LN, PIIIP, and serum IL-1β, TNF-α, hs-CRP, MMP-9, and other inflammatory factors in patients with CHF, and the degree of adverse reactions in the two groups was mild, which did not affect the clinical observations. There was no significant difference between the two groups, indicating that YQYYHXBXD combined with biomedicine medicine is safe and effective in the treatment of CHF and can improve clinical symptoms and cardiac function and inhibit ventricular remodeling. Its mechanism is related to anti-inflammatory effects and the regulation of serum sST2, galectin-3, LN, and PIIIP, and the inhibition of MF.

Huangqi Guizhi Wuwu Decoction and Shengmai Decoction (HQASM) (Wei et al., 2021) is composed of *A. membranaceus*, *P. quinquefolius*, *O. japonicus*, *S. miltiorrhiza*, *P. lactiflora*, *C. cassia*, *P. montana* var. *lobata*, *A. sinensis*, *Eupolyphaga sinensis*, *P. lactiflora*, *Z. officinale*, *Ziziphus jujuba* Mill. [Rhamnaceae; Ziziphi Jujubae Fructus] and *S. chinensis*. HQASM, on the basis of routine biomedicine medicine intervention, can reduce the levels of cTn-I, cTn-T, LDH and CK-MB in patients with diabetic cardiomyopathy and downregulate the levels of TGF-β1, MMP-2, IGF-1, IL-6, IL-1, TNF-α, NT-proBNP, sST2, and Gal-3, indicating that HQASM has anti-inflammatory and anti-MF effects in the treatment of DCM.

Shengmai powder and Danshen decoction (SMADS) consists of *A. membranaceus*, *S. miltiorrhiza*, *P. montana* var. *lobata*, *O. japonicus*, *S. chinensis*, *sandalwood*, *P. notoginseng*, *Wurfbainia villosa* var. villosa [Rubiaceae; Wurfbainiae Villosae Caulis], and *G. glabra* at a ratio of 30:30:30:9:6:6:6:6. Ultra-high performance liquid chromatography-quadrupole-time-of-flight mass spectrometry (UPLC-Q-TOF-MS/MS) was used to identify the components of Danshen Decoction *in vivo* and *in vitro*. A previous study (Shen and Sheng, 2022) revealed that, along with standard biomedicine medical treatments, Shengmai powder and Danshen decoction used for 2 months significantly reduced biomarkers such as hs-CRP, Lp-PLA2, GPM-140, PIIINP, LN, and HA, contributing to the prevention of MF progression. Table 8 summarizes clinical trials evaluating *S. miltiorrhiza*-based therapies for MF.

**TABLE 8:** *Salvia miltiorrhiza* metabolite and decoction combined with conventional biomedicine medicine to inhibit MF included in this study.

Metabolite/metabolite	Experimental type	Model	Mechanism of action	References
STS	RCT	PCI	The levels of CTGF, sST2, TGF-β1 and Gal-3 in serum of patients were downregulated	Deng (2023)
DHI	RCT	AMI	The levels of serum Gal-3, TGF-β1 and CTGF were downregulated, and the levels of NF-κB, cystatin C, MMP-9 and FGF-23 were decreased	Chen et al. (2019b)
FFDSDP	RCT	PCI	The levels of serum MMP-9, TGF-β1 and CTGF were downregulated	Xu et al. (2019)
QLQXC	RCT	CHF	Downregulation of serum NT-pro BNP, c TnT levels, downregulation of LN, HA, PCIII expression	Li and Sun (2023)
SMQXG	RCT	CHF	The levels of serum TGF-β1, MMP-2 and PIIINP were downregulated, and the mRNA and protein expressions of TLR4, MyD88 and NF-κB were decreased	Xu et al. (2024a)
SFYXD	RCT	HFrEF	The levels of serum sCD146, NT-proBNP, cTnI, Gal-3, Ang II, CgA and sST2 were downregulated, and the levels of PCO2, DO2 and VO2 were upregulated	Wang et al. (2024a)
YQHXD	RCT	CHF	Lp-PLA2, hs-CRP, IL-6, NT-proBNP, ECV and T1 values were downregulated	Zhang et al. (2023a)
SGYXD	RCT	CHD	Reduce serum HA, PCIII levels	Gan et al. (2020)
SYYQHXD	RCT	CHF	The levels of plasma thrombin, TGF-β1, serum Col-I, Col-III and NT-proBNP were downregulated	Chu et al. (2020)
ZWBXD	RCT	CHF	The levels of serum PCI, PCIII, hyaluronic acid HA and LN were decreased	Yan (2019)
JWWDD	RCT	PCI	The levels of PCI, HA, PCIII and LN were decreased	Xie et al. (2018)
YQHYD	RCT	CHF	The serum levels of TGF-β1, CTGF, MMP-2, HA, PCIII, LN and PCI were decreased	Cui et al. (2017)
YQYYHXD	RCT	CHF	The levels of serum IL-1β, hs-CRP, TNF-α, MMP-9, TGF-β1, PCIII, sST2 and Galectin-3 were decreased	Lu et al. (2022a)
BXHJ	RCT	CHF	The plasma levels of IL-1β, TNF-ɑ, IL-6, CRP, sST2 and Gal-3 were decreased	Bai et al. (2021)
YQYYHBXD	RCT	CHF	The levels of serum sST2, Galectin-3, LN, PIII, IL-1β, TNF-α, hs-CRP and MMP-9 were downregulated	Zhang and Wei (2021)
HQASM	RCT	CHF	The levels of TGF-β1, MMP-2, IGF-1, IL-6, IL-1, TNF-α, NT-proBNP, sST2 and Gal-3 were downregulated	Wei et al. (2021)
SMADS	RCT	Diabetic cardiomyopathy	The contents of hs-CRP, Lp-PLA2, GPM-140, PIIINP, LN and HA were downregulated	Shen and Sheng (2022)

## 6 The combined effects of *Salvia miltiorrhiza* Bunge with other preparations

Research has shown that the extracts of *S. miltiorrhiza* and *C. Tinctorius* (SCE) constitute a standardized preparation composed of two TCM botanical drugs, *S. miltiorrhiza* and *C. Tinctorius*. Its main pharmacologically active metabolites are phenolic acids, diterpenes, and flavonoids, such as sal B, Tan IIA, and hydroxysafflor yellow A. SCE is widely used to relieve angina in patients with coronary heart disease (Zhang et al., 2015). Compared with the control (Yang et al., 2019) SCE administration *in vivo* significantly increased the survival rate of mice after MI; inhibited myocardial inflammation; reduced the levels of H3K4 trimethylation (H3K4me3) and H3K36 trimethylation (H3K36me3) in the Smad3 promoter; and inhibited the expression of CoL-I, CoL-IIII and Sma RNA and protein, indicating that the reduction in MF caused by SCE was related to the downregulation of TGF-β1/Smad3 signal transduction. To confirm synergy between SCE, studies comparing SCE to each component alone are needed.

The TCM formulas *P. quinquefolius* and *S. miltiorrhiza* (PS). PS is reported to inhibit inflammatory responses and reduce endothelial damage. However, current data lack direct comparisons between individual and combined effects, limiting conclusions on true synergy. Research has shown that the concentrated granule preparation of PS at a ratio of 10:30 can be used to identify 223 metabolites through liquid chromatography, 147 of which were identified via positive ion mode and 76 of which were identified via negative ion mode. After gavage administration of PS at different doses (3 g/kg/d and 9 g/kg/d) for 4 weeks (Li X. et al., 2023), PS improved cardiac structure and function in rats with AMI, reduced infarct size, alleviated inflammatory cell infiltration, increased the expression of HGF and bFGF in the serum, and increased the levels of MVD and CD31 in myocardial tissues. PS reduced MF via miR-155-5p/HIF-1α/VEGF pathway inhibition. Whether this effect is synergistic requires validation through dose-response comparisons of individual vs. combined treatments.

*A. membranaceus* and *S. miltiorrhiza* (AS) are among the most popular phytomedicines in the world. They can also improve blood circulation in mice with myocardial infarction, protect against ischemia-reperfusion injury, and enhance cardiac function. Increasing evidence suggests that AS have protective effects against myocardial infarction. In accordance with the production standards for TCM formula granules, the granules of *AS* are prepared through processes such as decoction, filtration, concentration, drying, mixing, and granulation. Mice with MI were administered aqueous extracts of AS by gavage at a dose of 2 mL/kg, which is equivalent to 1.875 g/kg Astragalus and 0.9375 g/kg *S. miltiorrhiza*. After 6 weeks of treatment, compared with the model group, the intervention group presented reduced expression of COL-I and MMP9 induced by MI to some extent, as verified by tissue staining and protein analysis. These findings indicate that AS may inhibit MF, but synergistic mechanisms remain unconfirmed due to the absence of comparative data on individual herb contributions (Zhang M. X. et al., 2022).

A network pharmacology - based investigation uncovered the common and unique biological processes of *A. membranaceus* (HQ) and *S. miltiorrhiza* radix et rhizoma (DS) in CHD. Compared with the HQ or DS monotherapy groups, the HQ + DS combination treatment group presented significantly increased LVEF and LVFS values. Additionally, the infarct size and degree of fibrosis were significantly reduced, and the levels of lipid metabolism and blood viscosity indicators were markedly decreased. In terms of cardiac function parameters, the LVEDd, LVEDs, and CSA were significantly lower in both the HQ monotherapy group and the HQ + DS group than in the MI group. Compared with those in the MI group, coagulation indicators (APTT, PT, TT, FIB) were significantly lower in both the DS monotherapy group and the HQ + DS treatment group. This study revealed that HQ has specific advantages in alleviating cardiac remodeling, whereas DS has specific advantages in regulating hypercoagulability. These findings indicate that HQ and DS demonstrate complementary actions in treating CHD. Synergistic effects require further validation through isobolographic analysis or similar methods to quantify interaction effects (Zhang et al., 2021).

Zhang et al. (2024) identified 30 active metabolites and 41 common targets related to MF for the botanical drug pair *S. miltiorrhiza* and *P. montana* var. *lobata* through databases such as GeneCards and STRING. KEGG pathway analysis revealed 73 significantly enriched pathways, among which 6 were related to MF. The “active metabolite-target-pathway” network revealed that active metabolites such as luteolin from *S. miltiorrhiza*, Tan IIA from *S. miltiorrhiza*, puerarin from Pueraria lobata, and β-sitosterol from Pueraria lobata can regulate lipid metabolism and atherosclerosis, the AGE-RAGE pathway in diabetic complications, shear stress and atherosclerosis, and the HIF-1, TNF, and IL-17 pathways to exert anti-MF effects. These findings provide a theoretical basis and new ideas for further research on the mechanism of action of the *S. miltiorrhiza* and Pueraria lobata botanical drug pair in treating MF. The network suggests potential anti-MF mechanisms of *S. miltiorrhiza* and Pueraria lobata metabolites. However, synergistic interactions between these metabolites require experimental validation (e.g., checkerboard assays).

## 7 Conclusion and future prospects

**FIGURE 9:**
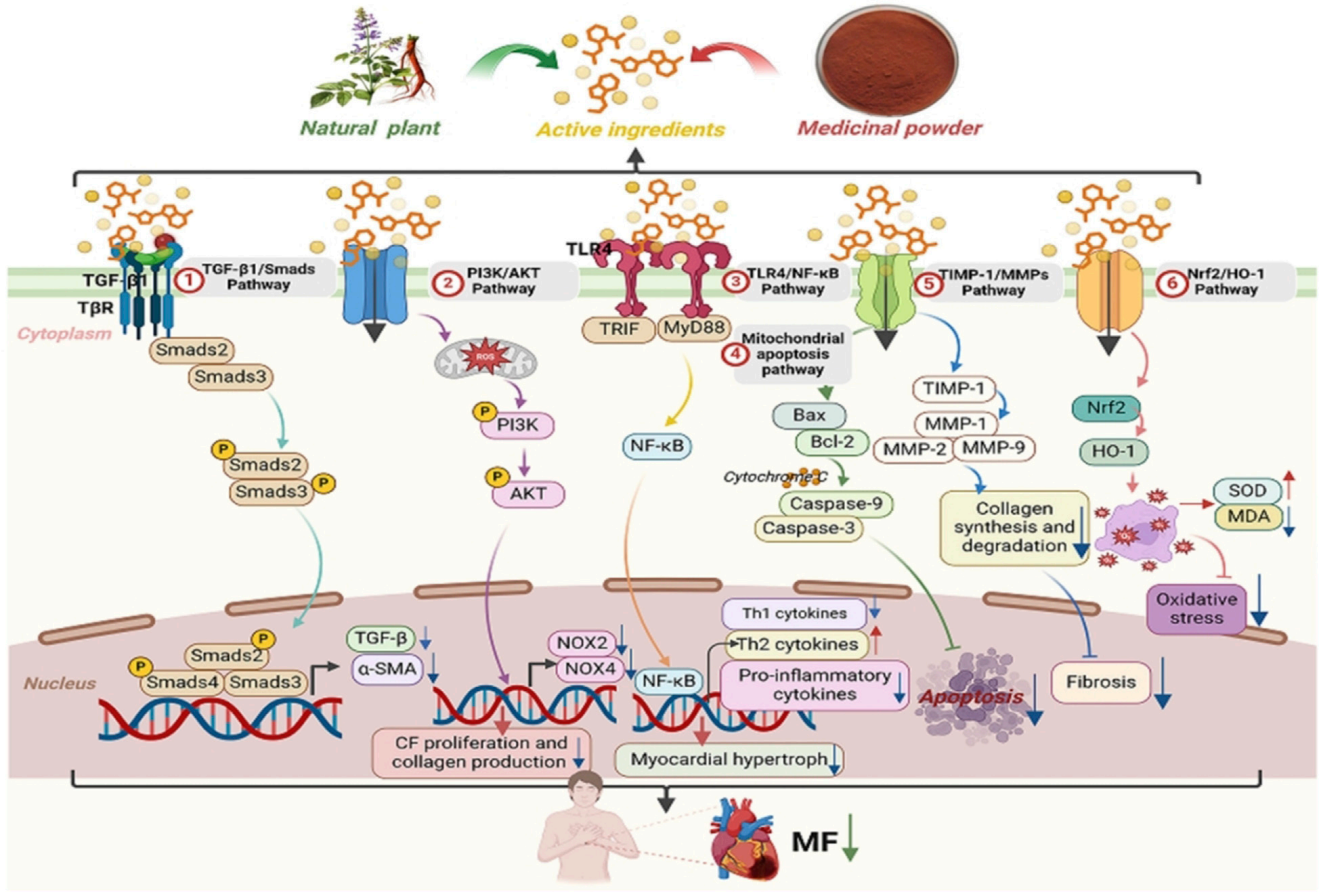
Mechanism of *Salvia miltiorrhiza* Bunge in the treatment of MF (① SM inhibitsMFby downregulating TGF-β1, Smad2/3, and their phosphorylation levels; ② SM inhibits MF by downregulating the phosphorylation levels of PI3K, Akt, and mTOR proteins; ③ SM inhibits MF by downregulating the expression of TLR4 and the activation of downstream TAK1 and NF-κB; ④ SM inhibits apoptosis and MF by upregulating the expression of Bax, Caspase-3, Caspase-9, and cytochrome C, and downregulating Bcl-2 protein; ⑤ SM regulates the TIMP-1/MMPs pathway to inhibit MF by downregulating the expression of MMP2 and MMP9, and upregulating TIMP-1 expression; ⑥ SM regulates the Nrf2/HO-1 signaling pathway to inhibit MF by upregulating the expression of antioxidant proteins such as Nrf2 and HO-1).

MF in cardiovascular diseases has attracted considerable attention, but effective drugs to reverse MF are lacking. Despite significant advancements, no approved drugs exist for reversing MF that can effectively reverse MF. Various active metabolites of *S. miltiorrhiza*, along with their formulated preparations, exhibit considerable efficacy in mitigating MF and enhancing cardiac function. This evidence highlights the strong ability of *S. miltiorrhiza* to prevent and manage MF. *S. miltiorrhiza* is a promising plant for research as a candidate drug in the prevention of pulmonary fibrosis; thus, it offers significant potential for the development of novel antifibrotic therapy. In this article, we examined advancements in research concerning the active metabolites of *S. miltiorrhiza* and its formulations in the context of anti-MF. These metabolites can significantly modulate immune inflammatory damage and prevent cardiomyocyte apoptosis by regulating various mechanisms, including the PI3K/AKT signaling pathway, the NF-κB signaling pathway, the modulation of TGF-β1 expression, and the upregulation of the JNK signaling pathway (Table 9) (Figure 9). Consequently, these actions demonstrate beneficial effects in the treatment of anti-MF. To summarize, TCM has demonstrated applicability across various stages of treatment for fibrosis and associated cardiovascular diseases owing to its multimetabolite, multitarget, and multilevel attributes. Furthermore, TCM has benefits in enhancing fibrosis in various organs, suggesting a novel approach for the development of antifibrotic pharmacological agents.

**TABLE 9:** The mechanisms of action of the main active metabolites of *Salvia miltiorrhiza* metabolite in improving MF.

Active metabolite	Molecular formula	Molecular weight	Model	Mechanism	References
Tan IIA	C_19_H_18_O_3_	294.34	ISO	Reduced expression of p-PI3K and p-Akt proteins	Song et al. (2022)
			ISO	Reduced expression of p-PI3K/PI3K and p-AKT/AKT proteins	He Dequan and Wang (2022)
			—	Regulating the PKA/CREB pathway boosts MMP-1 synthesis but reduces MMP-2 and MMP-9 levels	Mao et al. (2014)
			AAC	The levels of HYP and NF-κB p65 protein are decreased	Cai et al. (2014)
			LAC	NADPH oxidase and SOD activity is reduced	Yuan et al. (2019)
			LAC	HYPα-SMA, TGF-β1, HO-1, and MDA levelswere downregulated	Cai et al. (2016)
			LAD	TGF-β, α-SMA, MMP2, and MMP9 mRNA levels are decreased, while SOD activity is elevated	Chen et al. (2021)
			Ang II	Downregulation of MMP-9, MMP-2, TGF-β1, p-Smad2/3, SP-1 and caspase-3, caspase-9 protein	Chen et al. (2017b)
			Ang II	The rate of cell proliferation, along with the expression levels of Hyp, Col-I, Col-III, and TIMP-2 mRNA, exhibited a downregulation	Wang et al. (2019b)
			Ang II	The rate of proliferation of CFs was suppressed, and there was a reduction in the expression levels of Col-I	Fu and Sun (2016)
			Ang II	Suppress the proliferation rate of CFs and decrease the levels of ROS.	Chan et al. (2011)
Sal B	C_36_H_30_O_16_	718.61	ISO	Lower TGF-β1, Smad2, and Smad3, and boost Smad7	Gao et al. (2019)
			STZ/HUVEC	Regulate the PI3K/AKT pathway	Li et al. (2020)
			ISO	PI3K, AKT, p-AKT, and mTOR levels were reduced	Du et al. (2019)
			STZ	Inhibit TGF-β1 signaling pathway by up-regulating Smad 7	Luo et al. (2023)
			High glucose induction	The proliferation of CFs was inhibited, and the protein levels of α-SMA, β-catenin and p-GSK 3β were decreased	Wang et al. (2020)
			Ang II	The abnormal proliferation and Hyp content of CFs were decreased, and the expression of Col I and α-SMA protein was downregulated	Huang et al. (2017)
			TGF-β1	Inhibition of CFs proliferation rate	Luo et al. (2014)
			MMP-9	Inhibit the transformation of cardiac fibroblasts into myofibroblasts phenotype	Wang et al. (2011)
			STZ + 30% high fat diet feeding	Inhibit the proliferation of fibroblasts, reduces the activation of the TGF-β/Smad3 signaling pathway	Lu (2024)
			Ang II	Downregulation of Galectin-3, TGF-β, and Smad3 protein expression in CFs	Sun (2017)
STS	C_10_H_17_NaO_6_S	396.39	Ang II	Boost MMP-1expression and activity	Yang et al. (2009)
			Ang II	Inhibited ROS production	Yang et al. (2009)
			RCT	The levels of CTGF, sST2, TGF-β1 and Gal-3 in serum of patients were downregulated	Deng (2023)
			Ang II	Myocardial tissue exhibits reduced CoL-I and CoL-III, along with lower levels of Keap1, cytoplasmic Nrf2 protein, and MDA.	Li et al. (2018a)
CTS	C_19_H_20_O_3_	296.36	STZ	Inhibition of STAT 3 pathway	Lo et al. (2017)
			ISO	Regulate MMP-2	Ma et al. (2012a)
			Ang II	Inhibiting the phosphorylation of extracellular signal-regulated kinase 1/2 and the expression of COX-2, NOX-2 and NOX-4 induced by Ang II	Ma et al. (2014)
SAA	C_9_H_9_O_5_ · Na	220.15	LAD	In myocardial tissue, SOD activity and collagen volume fraction decreased, while MDA levels increased	Liu and Wang (2021)
Sal A	C_26_H_22_O_10_	494.45	DOX	Reduce the expression of Galectin-3 and TGF-β/Smads proteins in myocardial tissue	Ai et al. (2022)
			High glucose induction	Inhibited the expression of TGF-β1 and β-catenin, while upregulating the expression of p-GSK-3β, inhibited the proliferation of CFs	Han et al. (2022)

### 7.1 Critical limitations in current research

Despite extensive preclinical studies on *S. miltiorrhiza* for MF prevention, the precise material basis of its therapeutic effects remains unclear. The scientific exploration faces critical bottlenecks requiring urgent breakthroughs: (1) Incomplete Elucidation of Material Basis and Molecular Mechanisms and Targeted Mechanisms:Although the multitarget properties of *S. miltiorrhiza* and its metabolites have been preliminarily confirmed, systematic validation of structure-function relationships for key bioactive components (e.g., Tan IIA, Sal B) is lacking. For instance, whether Tan IIA’s regulation of the TGF-β/Smad pathway exhibits dose-dependency remains unclear, and most studies rely on single cell lines (e.g., H9c2 cardiomyocytes), raising concerns about reproducibility across diverse pathological models. This limits the accurate quantification of “multi-component-multi-target” effects. (2) Limitations in Preclinical Model Translatability in Preclinical Models:Current research excessively depends on simplified models, including static *in vitro* cardiac fibroblasts (lacking mechanical stress and intercellular interactions) and rodent models (where MF induced by myocardial infarction differs significantly from human chronic fibrosis). For example, rodents’ robust cardiac regenerative capacity may underestimate *S. miltiorrhiza*’s long-term antifibrotic effects, while *in vitro* systems fail to recapitulate dynamic immune cell infiltration in human myocardial microenvironments, hindering extrapolation to complex human pathophysiology. (3) Suboptimal Clinical Trial Methodologies:Existing clinical trials suffer from insufficient statistical power (e.g., small sample sizes, n < 100, increasing type II error risks) and short follow-up periods (<6 months), which fail to capture the chronic nature of fibrosis progression. Improvements in LVEF may be overestimated due to placebo effects or natural disease fluctuations, and the absence of long-term safety data (e.g., hepatorenal toxicity) could obscure risks, impeding evidence accumulation for evidence-based medicine. (4) Absence of Quantitative Synergy Assessment for Herbal Formula Synergy:Combination therapies of *S. miltiorrhiza* with other herbs (e.g., *A. membranaceus*, *C. tinctorius*) show apparent synergy in experimental models (e.g., collagen deposition inhibition), but lack quantitative support from dose-response curves (DRC) and isobolographic analysis. The absence of combination index (CI) calculations leaves the distinction between “synergy” and “additive effects” hypothetical, severely restricting formula optimization and standardization. (5) Real-World Evidence Shortcomings:Available clinical data primarily derive from single-center observational studies, lacking integration of multi-dimensional real-world evidence (RWE) platforms incorporating electronic health records (EHR), dynamic biomarkers (e.g., wearable HRV monitoring), and genomic data (e.g., fibrosis-related SNPs). This limits the optimization of personalized treatment protocols.

### 7.2 Future prospects

Future research directions for *S. miltiorrhiza* in MF: integrative approaches and technological innovations. To address the aforementioned bottlenecks, future research must prioritize interdisciplinary integration and technological innovation, establishing a “basic-translational-clinical” trinity research paradigm. The following critical pathways warrant immediate exploration:Multi-Omics Integrated Mechanistic Exploration: (1) Integrate spatial metabolomics and single-cell proteomics to map spatiotemporal variations in *S. miltiorrhiza* bioactive components within tissue microenvironments. For example, concentration gradients of Sal B in ischemic vs. non-ischemic cardiomyocytes may modulate targeting efficiency—a gap in data currently preventing precise modeling of “multi-component-multi-target” effects. Machine learning algorithms (e.g., random forest, deep neural networks) can predict three-dimensional regulatory relationships among components-targets-pathways, providing a theoretical framework for precision drug design. (2) Precision Humanized Pathological Model Development: Differentiate induced pluripotent stem cell (iPSC)-derived functional cardiomyocytes and cardiac fibroblasts, then construct dynamic three-dimensional cultures using Organ-on-a-Chip technology to recapitulate human MF microenvironments (hypoxia, mechanical stress, inflammatory gradients). These models overcome species disparities, enabling clinically predictive preclinical evaluations of drug responses and toxicities. (3) Methodological Enhancements for Evidence-Based Practice:Design multi-center, large-sample (n ≥ 500) double-blind RCTs with composite endpoints (e.g., fibrosis area via cardiac MRI, dynamic serum biomarkers PIIINP/Galectin-3) instead of single metrics. Extend follow-up to ≥2 years to capture long-term efficacy/safety signals. Apply Bayesian statistical models to quantify efficacy-risk ratios and develop dynamic risk prediction scores (e.g., FIBRO-SCORE). (4) Systems Pharmacology Validation of Synergy:Quantitatively validate herbal formula synergy using Chou-Talalay Combination Index (CI) models, Bliss independence analysis, isobolographic assays, and checkerboard experiments to characterize interaction patterns (synergy/addition/antagonism). Track pharmacokinetic-pharmacodynamic (PK/PD) correlations of key components via targeted metabolomics to resolve molecular thresholds and spatiotemporal specificity of synergy. (5) Real-World Data-Driven Precision Therapeutics:A causality-oriented real-world evidence (RWE) platform should integrate longitudinal genomic data (e.g., fibrosis-related SNP dynamics), high-dimensional proteomic data (e.g., single-cell extracellular matrix remodeling markers), and wearable device-monitored parameters (e.g., heart rate variability [HRV], daily activity). Marginal structural models (MSM) must control time-varying confounders, while target trial emulation frameworks should validate causal effects of Salvia interventions, mitigating selection bias in observational studies. This will advance the transition from “population-based therapy” to “individualized intervention.”

*S. miltiorrhiza*, as the interface between traditional medical wisdom and the paradigm of modern precision medicine, holds great potential for antifibrotic applications. The full realization of this potential relies on the integration of interdisciplinary technologies and the establishment of a global collaborative research network. This endeavor necessitates the convergence of multi-omics technologies, artificial intelligence, and evidence-based medicine of multi-omics technologies, artificial intelligence, and evidence-based medicine, as well as the creation of data-sharing mechanisms and standardized research frameworks to accelerate the construction of the translational medicine value chain. Only through systematic validation via multidimensional evidence chains can this traditional medicinal plant establish a precise therapeutic strategy for the prevention and treatment of myocardial fibrosis that transcends time and space.

## Glossary

**MF**: Myocardial fibrosis
**TCM**: Traditional Chinese medicine
**CFs**: cardiac fibroblasts
**PI3K**: Phosphatidylinositol3 kinase
**TGF-β**: Transforming Growth Factor - β
**NF-κB**: Nuclear factor kappa-B
**RAAS**: Renin-Angiotensin-Aldosterone System
**MMPs**: Matrix metalloproteinases
**ACEI**: angiotensin-converting enzyme inhibitors
**ARB**: angiotensin II receptor antagonist
**ARNI**: Angiotensin Receptor-Neprilysin Inhibitor
**AAC**: Abdominal Aortic Constriction
**SGLT2i**: Sodium-Glucose Cotransporter 2 Inhibitors
**α-SMA**: alpha-smooth muscle actin
**ECM**: extracellular matrix
**Tan IIA**: Tanshinone IIA
**ISO**: isoprenaline
**Col-I**: Collagen type I
**Col-III**: Collagen type III
**CHF**: Chronic Heart Failure
**Ang II**: Angiotensin II
**BNP**: Brain Natriuretic Peptide
**LVMI**: Left Ventricular Mass Index
**LVM**: Left Ventricular Mass
**Sal B**: *Salvianolic* acid B
**STZ**: Streptozotocin
**AKT**: protein kinase B
**ESZWD**: Er Shen Zhen Wu Decoction
**QSYQDP**: Qishen Yiqi Dropping Pills
**CFDA**: China Food and Drug Administration
**Sal A**: Salvianolic acid A
**TNF-α**: Tumor Necrosis Factor-α
**Hcy**: Homocysteine
**ET**: Endothelin
**HQBXD**: Huangqi Baoxin Decoction
**QSLWFG**: Qi Shen Liu Wei formula granules
**KXP**: Kuo Xin Prescription
**SQJXP**: Shenqi Jianxin Prescription
**YXFTY**: Yixin Futing Yin
**CHF**: chronic heart failure
**YQYYHXTLP**: Yiqi Yangyin Huoxue Tongluo Prescription
**DCM**: Dilated Cardiomyopathy
**QLQXC**: Qiliqiangxin capsule
**SSY**: Sanshen Yin
**QSG**: Qishen Granule
**SSYXC**: Shensongyangxin Capsule
**FZHYP**: Fuzheng Huayu Prescription
**TIMPs**: tissue inhibitors of metalloproteinases
**TIMP-1**: Tissue inhibitor of metalloproteinase-1
**ECM**: extracellular matrix
**FZHYC**: Fuzheng Huayu Capsule
**YQHXC**: Yiqi Huoxue metabolite
**ZGJTP**: Zuo Gui Jiang Tang Shu Xin Prescription
**YXTMP**: Yangxin Tongmai Prescription
**QSYQP**: Qi Shen Yi Qi Prescription
**LAD**: Left Anterior Descending Branch
**CFZZTZC**: metabolite Zhenzhu Tiaozhi Capsule
**iNOS**: inducible nitric oxide synthase
**NO**: nitric oxide
**6 mwd**: 6-min walk distance
**HFpEF**: heart failure and preserved ejection fraction
**HF**: heart failure
**SMQXG**: Shengmai Qiangxin Granules
**BSHXD**: Bushen Huoxue Decoction
**YXT**: Yixintai
**DHI**: Danhong injection
**MI**: myocardial infarction
**ROS**: reactive oxygen species
**RNS**: reactive nitrogen species
**SAA**: Salvianic acid A
**SOD**: superoxide dismutase
**MDA**: malondialdehyde
**STS**: Sodium Tanshinone IIA Sulfonate
**Hyp**: Hydroxyproline
**CDSDP**: metabolite Danshen Dripping Pills
**GXV**: Guanxin V
**GXT**: Guanxintai
**DXTMG**: Danxiong Tongmai Granules
**NO-SMS**: newly optimized formulation of Shengmaisan
**NT-ProBNP**: N-Terminal Pro-B-Type Natriuretic Peptide
**AIM**: Autoimmune Myocarditis
**COA**: Coarctation of the Aorta
**YXT**: Yixintai
**HQQR**: Huoxue Qianyang Qutan Recipe
**YXTG**: Yixintai Granules
**YQHXD**: Yiqi Huoxue Decoction
**FZHYD**: Fuzheng Huayu Decoction
**JSF**: Jiashenfang
**BSHXD**: Busheng Huoxue Decoction
**RCT**: randomized controlled trial
**SMQXG**: Shengmai Qiangxin Granules
**SFYXD**: Shenfu Yixin Decoction
**HFrEF**: Heart Failure with Reduced Ejection Fraction
**YQHXP**: Yiqi Huoxue prescription
**SGYXP**: Shugan Yixin prescription
**SYYXHXD**: Shenyuan Yiqi Huoxue Decoction
**ZWBXD**: Zhenwu Baoxin Decoction
**JWWDD**: Jiawei Wendan Decoction
**YQHYD**: Yiqi Huayu Decoction
**YQYYHXD**: Yiqi Yangyin Huoxue Decoction
**YQYYHXBXD**: Yiqi Yangyin Huoxue Buxin Decoction
**HQASM**: Huangqi Guizhi Wuwu Decoction and Shengmai Decoction
**SMADS**: Shengmai Powder and Danshen Decoction
***A. membranaceus***: *Astragalus membranaceus* (Fisch.) Bunge [Fabaceae; Astragali Radix]
***P. ginseng***: *Panax ginseng* C.A. Mey. [Araliaceae; Ginseng Radix et Rhizoma]
***A. carmichaelii***: *Aconitum carmichaelii* Debx. [Ranunculaceae; Aconiti Lateralis Praeparata]
***D. sophia***: *Descurainia sophia* (L.) Webb ex Prantl [Brassicaceae; Descurainiae Semen]
***C. cassia***: *Cinnamomum cassia* Presl [Lauraceae; Cinnamomi Cortex]
***C. tinctorius***: *Carthamus tinctorius* L. [Asteraceae; Carthami Flos]
***M. officinalis***: *Magnolia officinalis* Rehd. et Wils. [Magnoliaceae; Magnoliae Cortex]
***C. reticulata***: *Citrus reticulata* Blanco [Rutaceae; Citri Reticulatae Pericarpium]
***P. ginseng***: *Panax ginseng* C.A. Meyer [Araliaceae; Ginseng Radix Rubra]
***A. macrocephala***: *Atractylodes macrocephala* Koidz.[Asteraceae; Atractylodis Macrocephalae Rhizoma]
***E. brevicornu***: *Epimedium brevicornu* Maxim.[Berberidaceae; Epimedii Folium]
***P. cocos***: *Poria cocos* (Schw.) Wolf [Polyporaceae; Poria]
***C. cassia***: *Cinnamomum cassia* Presl [Lauraceae; Cinnamomi Ramulus]
***C. anthriscoides***: *Conioselinum anthriscoides* (Chuanxiong) [Apiaceae; Ligustici Chuanxiong Rhizoma]
***P. heterophylla***: *Pseudostellaria heterophylla* (Miq.) Pax [Caryophyllaceae; Pseudostellariae Radix]
***O. japonicus***: *Ophiopogon japonicus* (Thunb.) Ker Gawl. [Asparagaceae; Ophiopogonis Radix]
***R. glutinosa***: *Rehmannia glutinosa* (Gaertn.) Libosch. ex DC. [Scrophulariaceae; Rehmanniae Radix]
***S. chinensis***: *Schisandra chinensis* (Turcz.) Baill. [Schisandraceae; Schisandrae Fructus]
***G. glabra***: *Glycyrrhiza glabra* L. [Fabaceae; Glycyrrhizae Radix et Rhizoma]
***P. notoginseng***: *Panax notoginseng* (Burkill) F.H.Chen [Araliaceae; Notoginseng Radix et Rhizoma]
***P. lactiflora***: *Paeonia lactiflora* Pall. [Paeoniaceae; Paeoniae Radix Alba]
***A. carmichaelii***: *Aconitum carmichaelii* Debeaux [Ranunculaceae; Aconiti Lateralis Praeparata]
***L. japonica***: *Lonicera japonica* Thunb. [Caprifoliaceae; Lonicerae Japonicae Flos]
***P. montana* var. lobata**: *Pueraria montana* var. lobata (Willd.) Maesen & S.M.Almeida ex Sanjappa & Predeep [Fabaceae; Puerariae Lobatae Radix]
***R. glutinosa***: *Rehmannia glutinosa* (Gaertn.) Libosch. ex DC. [Scrophulariaceae; Rehmanniae Radix]
***C. officinalis***: *Cornus officinalis* Siebold & Zucc. [Cornaceae; Corni Fructus]
***P. vulgaris***: *Prunella vulgaris* L. [Lamiaceae; Prunellae Spica]
***J. regia***: *Juglans regia* L. [Juglandaceae; Juglandis Semen]
***P. massoniana***: *Pinus massoniana* Lamb. [Pinaceae; Pinus Massoniana Pollen]
***G. pentaphyllum***: *Gynostemma pentaphyllum* (Thunb.) Makino [Cucurbitaceae; Gynostemmatis Pentaphylli Herba]
*C. chinensis*: *Coptis chinensis* Franch. [Ranunculaceae; Coptidis Rhizoma]
***C. monogyna***: Crataegus monogyna Jacq. [Rosaceae; Crataegi Fructus]
***C. ×aurantium* f. aurantium**: *Citrus × aurantium* f. aurantium [Rutaceae; Aurantii Fructus Immaturus]
***B. sacra***: *Boswellia sacra* Flück. [Burseraceae; Boswelliae Resina]
***C. myrrha***: *Commiphora myrrha* (T.Nees) Engl. [Burseraceae; Myrrha]
***P. kurroa***: *Picrorhiza kurroa* Royle ex Benth. [Scrophulariaceae; Picrorhizae Rhizoma]
***A. bidentata***: *Achyranthes bidentata* Blume [Amaranthaceae; Achyranthis Bidentatae Radix]
***A. gramineus***: *Acorus gramineus* Aiton [Acoraceae; Acori Graminei Rhizoma]
***R. multiflora***: *Reynoutria multiflora* (Thunb.) Moldenke [Polygonaceae; Reynoutriae Multiflorae Caulis]
***C. yanhusuo***: *Corydalis yanhusuo* (Y. H. Chou & Chun C. Hsu) W. T. Wang ex Z. Y. Su & C. Y. Wu [Papaveraceae; Corydalis Rhizoma]
***L. chinense***: *Lycium chinense* Mill. [Solanaceae; Lycii Fructus]
***E. senticosus***: *Eleutherococcus senticosus* (Rupr. & Maxim.) Maxim. [Araliaceae; Acanthopanacis Senticosi Radix et Rhizoma]
***T. crispa***: *Tinospora crispa* (L.) Hook.f. & Thomson [Menispermaceae; Tinosporae Caulis]
***E. japonicum***: *Eupatorium japonicum* Thunb. [Asteraceae; Eupatorii Herba]
***W. indica***: *Waltheria indica* L. [Sterculiaceae; Waltheriae Indicae Herba]
***C. Ostreae***: *Concha Ostreae* [Ostreidae; Concha Ostreae]
***U. rhynchophylla***: *Uncaria rhynchophylla* (Miq.) Miq. [Rubiaceae; Uncariae Ramulus cum Uncis]
***C. zedoaria***: *Curcuma zedoaria* (Christm.) Roscoe [Zingiberaceae; Curcumae Zedoariae Rhizoma]
***C. aromatica***: *Curcuma aromatica* Salisb. [Zingiberaceae; Curcumae Aromatica Rhizoma]
***D. costus***: *Dolomiaea costus* (Falc.) Kasana & A.K.Pandey [Asteraceae; Dolomiaeae Costus]
***L. chinense***: *Lycium chinense* Mill. [Solanaceae; Lycii Fructus]
***C. deserticola***: *Cistanche deserticola* Ma [Orobanchaceae; Cistanches Herba]
***Z. officinale***: *Zingiber officinale* Roscoe [Zingiberaceae; Zingiberis Rhizoma]
***P. asiatica***: *Plantago asiatica* L. [Plantaginaceae; Plantaginis Semen]
***A. catechu***: *Areca catechu* L. [Arecaceae; Arecae Semen]
***R. rosea***: *Rhodiola rosea* L. [Crassulaceae; Rhodiolae Radix et Rhizoma]
***V. negundo***: *Vitex negundo* L. [Verbenaceae; Viticis Negundi Herba]
***B. chinense***: *Bupleurum chinense* DC. [Apiaceae; Bupleuri Radix]
***M. oleifera***: *Moringa oleifera* Lam. [Moringaceae; Moringae Oleiferae Folium]
***S. baicalensis***: *Scutellaria baicalensis* Georgi [Lamiaceae; Scutellariae Radix]
***P. armeniaca***: *Prunus armeniaca* L. [Rosaceae; Armeniacae Semen]
***C. chinensis***: *Coptis chinensis* Franch. [Ranunculaceae; Coptidis Rhizoma]
***A. vera***: *Aloe vera* (L.) Burm.f. [Asphodelaceae; Aloes]
***T. kirilowii***: *Trichosanthes kirilowii* Maxim. [Cucurbitaceae; Trichosanthis Fructus]
***S. glabra***: *Smilax glabra* Roxb. [Liliaceae; Smilacis Glabrae Rhizoma]
***P. quinquefolius***: *Panax quinquefolius* L. [Araliaceae; Panax Quinquefolii Radix]
***F. suspensa***: *Forsythia suspensa* (Thunb.) Vahl [Oleaceae; Forsythiae Fructus]
***T. aestivum***: *Triticum aestivum* L. [Poaceae; Triticus Aestivus]
***C. sativa***: *Cannabis sativa* L. [Cannabaceae; Cannabis Fructus]
***Z. jujuba***: *Ziziphus jujuba* Mill. [Rhamnaceae; Ziziphi Jujubae Fructus]
***W. villosa* var. villosa**: *Wurfbainia villosa* var. villosa [Rubiaceae; Wurfbainiae Villosae Caulis]
